# Direct Cell Reprogramming and Phenotypic Conversion: An Analysis of Experimental Attempts to Transform Astrocytes into Neurons in Adult Animals

**DOI:** 10.3390/cells12040618

**Published:** 2023-02-14

**Authors:** Rachel Dennison, Esteban Usuga, Harriet Chen, Jacob Z. Paul, Christian A. Arbelaez, Yang D. Teng

**Affiliations:** 1 Department of Physical Medicine and Rehabilitation, Harvard Medical School, Boston, MA 02129, USA; 2 Laboratory of SCI, Stem Cell and Recovery Neurobiology Research, Department of Physical Medicine and Rehabilitation, Spaulding Rehabilitation Hospital Network, Mass General Brigham, and Harvard Medical School, Boston, MA 02115, USA; 3 Neurotrauma Recovery Research, Department of Physical Medicine and Rehabilitation, Spaulding Rehabilitation Hospital Network, Mass General Brigham, and Harvard Medical School, Boston, MA 02115, USA

**Keywords:** astrocyte, direct cell conversion, direct cell reprogramming, functional multipotency of stem cells, neurodegeneration, neurotrauma, neuron

## Abstract

Central nervous system (CNS) repair after injury or disease remains an unresolved problem in neurobiology research and an unmet medical need. Directly reprogramming or converting astrocytes to neurons (AtN) in adult animals has been investigated as a potential strategy to facilitate brain and spinal cord recovery and advance fundamental biology. Conceptually, AtN strategies rely on forced expression or repression of lineage-specific transcription factors to make endogenous astrocytes become “induced neurons” (iNs), presumably without re-entering any pluripotent or multipotent states. The AtN-derived cells have been reported to manifest certain neuronal functions in vivo. However, this approach has raised many new questions and alternative explanations regarding the biological features of the end products (e.g., iNs versus neuron-like cells, neural functional changes, etc.), developmental biology underpinnings, and neurobiological essentials. For this paper per se, we proposed to draw an unconventional distinction between direct cell conversion and direct cell reprogramming, relative to somatic nuclear transfer, based on the experimental methods utilized to initiate the transformation process, aiming to promote a more in-depth mechanistic exploration. Moreover, we have summarized the current tactics employed for AtN induction, comparisons between the bench endeavors concerning outcome tangibility, and discussion of the issues of published AtN protocols. Lastly, the urgency to clearly define/devise the theoretical frameworks, cell biological bases, and bench specifics to experimentally validate primary data of AtN studies was highlighted.

## 1. Introduction

During early embryo development, neural stem cells (NSCs) proliferate and migrate to form the neural tube and enter neuronal, oligodendrocytic, and astrocytic differentiation in proper niches to build the central nervous system (CNS) [1,2]. In mammalian post-developmental brains, neurogenesis is confined to restricted regions (e.g., the subventricular zone of the lateral ventricles and the subgranular zone of the dentate gyrus in the hippocampus) [3]. Notably, these NSCs and neural progenitor cells (NPCs) appear insufficient to meet the migration and quantity requirements for producing new neurons to replace those lost to common neurotraumas or other disorders [4]. Thus, therapeutic neuronal regeneration in the adult mammalian CNS remains a perpetual challenge of modern biomedical research. In contrast to the peripheral nervous system (PNS), which possesses a comparatively high endogenous capacity for axonal regrowth, the mature CNS lacks the ability to fully regenerate axons lost in injuries or degenerative conditions [5]. To overcome these barriers, transplantations of neural tissues, neurons, NSCs, and/or NPCs have been extensively investigated to advance neurobiology and as potential therapeutic strategies [6,7].

**Figure 1:**
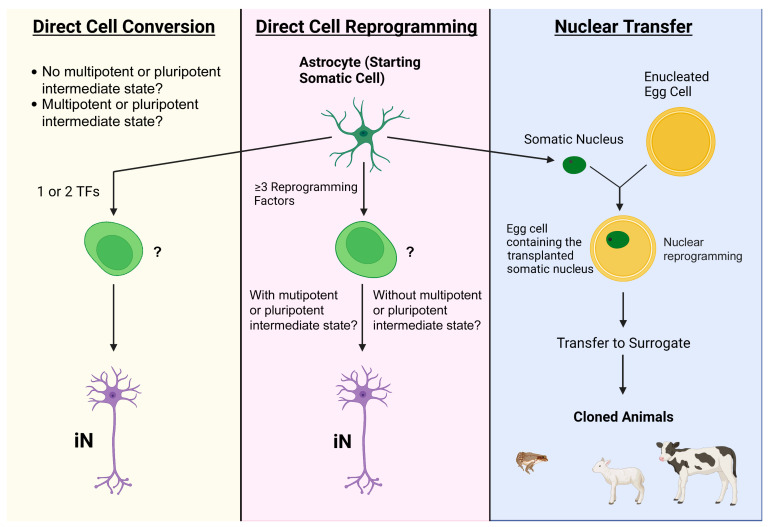
Summary of the formulas used in this paper to describe direct cell conversion and direct cell reprogramming relative to the somatic nuclear transfer presentation. For this review, we have tried to distinctively describe direct cell conversion and direct cell reprogramming as per the experimental methods utilized to initiate the process (see text for rationales and purposes). In the current research field, however, the two terms are interchangeably used. (**Left**) Direct cell conversion is used here to describe the change of one somatic cell type (e.g., astrocyte) into another (e.g., iN) via the intervention of 1–2 transcription factors (TFs), presumably without passing through a multipotent or pluripotent-like state. It was proposed by some investigators as a straightforward method to attain the desired cell type from an already differentiated cell. (**Middle**) Direct cell reprogramming is defined by the manipulation of ≥3 TFs (or other factors including small molecules, signaling pathway modulators, etc.) that affect multiple epigenetic and/or genetic elements. (**Right**) Somatic nuclear transfer, the original concept of nuclear/cell reprogramming, is a process through which the nucleus of the somatic cell is removed and transplanted into an enucleated egg cell/ovum that subsequently becomes a zygote before being transplanted into a surrogate or gestational carrier. The new-born animal will be a DNA clone of the somatic cell nucleus donor. Note: the same set of scientific questions remain to be answered for either direct cell conversion or direct cell programming, despite the difference between their initiation recipes (see text for more discussions).

Based on the novel mechanistic insights attained from major breakthroughs in research of conventional nuclear/cell reprogramming (i.e., somatic nuclear transfer) [8,9] and inducible pluripotent stem cells (iPSCs) [10], the rapidly evolving field of direct reprogramming or conversion-induced cell phenotypic change has been further enriched with endeavors trying to repair or regenerate the adult CNS by changing astrocytes into neurons in situ. Somatic nuclear transfer relies on the systematic nucleus reprogramming by egg cytoplasmic factors to generate zygotes and iPSC generation results from manipulation of pluripotency-specific genes to produce PSCs [8,9,10,11]. In contrast, the direct switch of cell phenotype has been proposed as the process of coaxing one kind of mature somatic cell into another, presumably without requiring the target cell to re-enter a multipotent or pluripotent developmental state. This has been shown to be feasible via forced expression or suppression of a few “pioneer” transcription factors (TFs) straightforwardly and/or through application of small molecules, micro-RNAs (miRs), or growth factors to affect signal transduction pathways that regulate phenotype-controlling genes (Figure 1) [12,13,14].

For neural repair purposes, it has been postulated that the AtN transition may be facilitated by the transiently heightened cell state and environment of pro-plasticity and pro-healing induced by injury or other pathological conditions. Such settings have been known to render cells more amenable to interventions that modify their epigenetic and genetic programs. However, to date, little attention has been given to analytically examining (1) potential differences between mature cell phenotypic conversion mediated by multiple TFs versus that through single or double TFs; (2) mechanistic underpinnings permitting “one step” phenotypic switch of somatic cells; (3) tumorigenesis risk of reprogramming neural cells in adult animals; and (4) efficiency of conserving the epigenetic signature (e.g., aging) of the target cell for neural recovery-related outcomes through applying the original nuclear/cell reprogramming principles [8,11]. In this review, our focus was on the assessment of published data from comprehensive basic science and translational AtN investigations to determine major challenges to this research field. There have been to date no endpoint data that meet conventional scientific standards to specify the intermediate and final epigenetic and genetic mechanisms resulting from single, double, or multiple TF(s)-induced phenotype change of cells in adult animals. Therefore, based on the original reports of the experimental methods utilized to set the transformation process in motion [12,13,14,15] and for this presentation per se, we proposed and used direct cell conversion to describe AtN procedures utilizing (or affecting) 1–2 TFs and direct cell reprogramming for those manipulating ≥ 3 TFs (or other multifactor formulas) relative to somatic nuclear transfer (i.e., global epigenetic modification). We anticipate the initiation recipe-framed subclassifications to trigger critical reactions, approaches to establishing specific mechanism-defined nomenclature systems, and efforts to develop more advanced investigations on the intermediate and final states of AtN cells following the first effect of 1, 2, or ≥3 TFs/other multifactor regimens.

## 2. Common Bench Approaches to Directly Attaining Neurons from Astrocytes

### 2.1. Transcription Factors

In 2008, Zhou, Melton, and colleagues published their first report of the direct reprogramming of murine pancreatic exocrine cells into insulin secreting cells that closely resembled pancreatic β-cells in vivo [15]. Using three AAV-delivered TFs (*Pdx1*, *Ngn3*, and *MafA*; note: see Supplementary Materials, Table S1, for definitions of all abbreviations and acronyms) that are pivotal to pancreatic development, β-cells were generated with a success rate of ~20%. Whereas pancreas/duodenum homeobox protein 1(Pdx1) was essential for early progenitor formation and Neurogenin 3 (Ngn3) was critical for endocrine lineage specification and differentiation, V-maf musculoaponeurotic fibrosarcoma oncogene homolog A (MAFA) was only detected in the adult mammalian pancreas where it was required for β-cell maturation [16]. The induced cells survived until the end of the study (i.e., 3 months) and were indistinguishable from endogenous cells in terms of size, morphology, and ultrastructure (e.g., insulin secreting granules), as well as the expression of key genes or proteins necessary for β-cell function (e.g., *GLUT2* or prohormone convertase and glucokinase). When the formula was applied to a streptozotocin-induced mouse model of type 1 diabetes, there was a significant and sustained improvement in insulin levels, fasting glucose levels, and glucose tolerance compared with the control group. However, glucose homeostasis was not completely restored, which was attributed to an insufficient number of induced cells when compared with wild-type animals, as well as the induced cells failing to form islets and remaining as individual and scattered cells frequently within acinar rosettes, which was indicative of their exocrine origin.

Similarly, the ectopic expression of 1–2 neurogenic TFs formed the molecular foundation of direct AtN conversion protocols. Aiming to promote more in-depth research explorations, we tried to draw a format distinction between direct cell reprogramming (i.e., manipulating ≥ 3 TFs) and direct cell conversion (i.e., affecting 1 or 2 TFs) in this review based on the original usages of the two terms [15,16,17,18,19,20,21,22]. Yet, these two phrases have been often used interchangeably in publications. More importantly, engineered expression of even a single TF may trigger multiple downstream activations of genes and signaling activities. It is therefore crucial for the field to systematically investigate all the possible intermediate states of the target cells that are under the effect of a given regimen of “reprogramming” or “conversion” treatment. Referencing the original definition of “nuclear/cell reprogramming” [8,9], the anticipated outcomes will likely enable making more precise conclusions regarding whether the impacted cells entered any pluripotent or multipotent phases prior to becoming the converted/reprogrammed cells to mitigate unnecessary confusion in theoretical and bench studies.

During neurodevelopment, astrocytic identity is in part established and maintained by the activation of astrocytic fate-determining genes at open chromatin sites and the closing of chromatin (indicative of transcriptional repression) at genetic loci relating to other lineages [17]. Indeed, developmental biology studies have identified a group of basic helix-loop-helix (bHLH) TFs called “proneural factors” represented by NeuroD1, Ascl1, and Neurogenin2 (Ngn2 or Neurog2) that are necessary and sufficient to initiate neurogenesis [18,19]. Due to their well-defined functions, no specific section is represented in this paper to detail their applications in AtN induction research. In essence, these TFs (e.g., NeuroD1, Ascl1, and Ngn2) are thought to re-model the astrocytic chromatin landscape by opening developmentally silenced pro-neural genetic loci to allow binding of secondary canonical factors that activate neuronal gene expression programs [18]. For example, Ascl1, as an on-target “pioneer factor,” may activate a chromatin switch from a starting-to-target cell, which could precede neuronal maturation and activate downstream TFs critical for neuronal development, including Zfp238, Sox8, and Dlx3 [19]. These proneural factors have also been reported as being effective to trigger AtN induction, yielding NeuN+ (i.e., mature-appearing) induced neurons (iNs; for common makers used to depict them, see Table S2) in a variety of brain regions sampled from different models (Table 1) [20,21,22,23]. However, the induction efficiency from using individual bHLH TFs (e.g., Ngn2/Neurog2) has varied substantially [24] and additional treatments such as BDNF, valproic acid, and Noggin were often required to produce iNs [25,26]. To improve experimental outcomes, protocols utilizing a combination of different factors and small molecules to improve AtN efficiencies are increasingly being applied (Table 1).

In general, strategies of administering a combination of TFs have been reported for achieving a greater level of transcriptional control via facilitating reprogramming at multiple checkpoints along the neurodevelopmental pathway. With a selection of TFs to be utilized (often through in situ hybridization screens), this approach was oriented to deliver a higher effect on producing iNs to promote functional recovery for in vivo models [27]. For example, when NeuroD1, Ascl1, LMX1A, and miR218 (decreasing *Onecut 2*/*OC-2* mRNA to promote neuronal features) were combined into the “NeA1218 cocktail” and injected into the brain of mice lesioned by 6-hydroxydopmaine (6-OHDA), a model of Parkinson’s disease, it was shown that the formula generated excitable dopaminergic iNs (~16%) from astrocytes that were linked to significantly improved motor behaviors (e.g., eradicating circling behavior caused by severe asymmetric loss of striatal dopamine) [26]. Of note, the treatment had no significant effect on amphetamine-induced circling behavior, which suggested that the amount of dopamine produced by the reprogrammed cells might be relatively low. Further, there were no data to confirm that the iNs directly released dopamine, possessed the cellular machinery to discharge dopamine, or produced other factor(s) which could be responsible for the observed functional improvement [26].

It has been cautioned that not all TFs drive the reprogramming or conversion process with the same potency and that adding more TFs to a cocktail might actually have deleterious effects on AtN induction efficiency by reducing the probability for the essential factors to interact with their targets [15]. Moreover, it appeared that the context, both the location and developmental stage, under which the TF was administered influenced the AtN process under the defined condition of each study. For example, ectopic expression of certain TFs (e.g., MAF bZIP Transcription Factor A: MAFA) beyond normal developmental contexts has been found to block cell differentiation [28]. Overall, these studies have illustrated the necessity of finetuning the choice of TFs, the dosage ratios of selected TFs, and the time windows in which to apply them before specific mechanisms of any phenotypic effect could be determined.

**Table 1:** The Common Bench Approaches for Direct AtN Conversion and Reprogramming.

Direct Cell Conversion Strategy	Induction Factor	In Vitro/In Vivo	Starting Cell Type/Animal Model	Vector/Delivery System	Induction Efficiency (%)	Phenotype
*Common Bench Approaches to AtN Conversion*						
Proneural factors/ pioneer transcription factors	Ascl1 (Mash1) [29]	In vitro	Dorsal midbrain astrocytes, WT mice (P5–P7)	Lentivirus	76.8 ± 6.4	Glutamatergic (19.4%), GABAergic (8/38 cells)
		In vivo	Dorsal midbrain astrocytes, WT mice (P60), M + F	AAV micropipette injection	92.1 ± 1.5	GABAergic (11.7 ± 4.0%), Glutamatergic (6.3 ± 1.3%)
	NeuroD1	In vivo	Cortical astrocytes, stab injury mouse model (P90–180), M + F [23]	AAV needle injection	90.6 ± 5.2	Glutamatergic, GABAergic
			Ischemic stroke model, GFAP-Cre × Rosa-YFP mice (adult), M [30]	Lentivirus stereotaxic injection	~66	Glutamatergic (~80%)
			Contusive SCI model T10 acute phase, WT mice (P60–P120), M + F [22]	Cre-FLEX AAV needle injection	~55	Glutamatergic
			Contusive SCI model T11-T12 chronic phase, WT mice (P60-P120), M + F [22]	Cre-FLEX AAV needle injection	>95	Glutamatergic
	Neurog2 (Ngn2)	In vivo [24]	Dorsal midbrain astrocytes, WT mice (adult)	AAV stereotaxic needle injection	96.3 ± 1.7	Glutamatergic (64.97 ± 8.04%), GABAergic (2.26 ± 2.07%)
			Dorsal horn T8–T10, WT mice (adult)		80.11 ± 5.42	Glutamatergic (50/9%), GABAergic (38.5%)
			Complete transection SCI model T8–T10, WT mice (adult)	AAV injection at L1–L2 dorsal surface	41.62 ± 22.82	Data not provided
	Dlx2	In vivo [31]	Striatal astrocytes, stab injury model, WT C57BL/6J mice (P60–P150), M + F	Retrovirus needle injection	~20 (30 dpi)	DCX+ immature neurons
			Striatal astrocytes in stab injury model, Aldh1l1-CreERT2 mice, (P60–P150), M + F	AAV9 needle injection	~70 (60 dpi)	MSN
	NeuroD1 + Dlx2	In vivo [27]	Striatal astrocytes, WT mice (P60–P140), M + F	rAAV2/5 stereotaxic injection	72.7	GABAergic (~85.0%), MSN (55.7%), interneurons (9.6%)
			Striatal astrocytes, R6/2 transgenic Huntington’s disease mouse model (P60–P150), M + F	rAAV2/5 stereotaxic injection	78.6	GABAergic, MSN, interneurons
			Striatum, YAC128 transgenic Huntington’s disease mouse model (middle aged, 15 months), M + F	rAAV2/5 stereotaxic injection	50.0	GABAergic, MSN, interneurons
	Ascl1 + Dlx2	In vivo [32]	Hippocampus, mesial temporal lobe epilepsy model, C57BL/6J mice (2–3 months), M	Retrovirus stereotaxic injection	70	GABAergic interneurons (~75%)
PTBP1 knockout	PTBP1	In vivo	Dentate gyrus, adult GFAP-CreERT2CAG-lox-stop-lox-tdTomato mice (5 months), M + F [33]	ASO-PTBP1 CSF injection	15 (2 mpi)	Granule cell layer neurons
			Dentate gyrus, aged GFAP-CreERT2CAG-lox-stop-lox-tdTomato mice (1 year), M + F [33]	ASO-PTBP1 CSF injection	5 (2 mpi)	Granule cell layer neurons
			Midbrain astrocytes, 6-OHDA Parkinson’s disease mouse model GFAP-Cre transgenic mouse [34]	AAV-shPTBP1	30–35 (12 wpi)	Dopaminergic
			Striatum of adult C57BL/6 mice (~P70), M [35]	AAV-GFAP-CasRx-Ptbp1 with gRNAs 5 + 6 targeting Ptbp1 stereotaxic injection	48.0 ± 10.0	Glutamatergic (~50%)
*Common Bench Approaches to AtN Reprogramming*						
Transcription factor and other reprogramming factor in combinations	NeAL218 * [26]	In vitro	Human midbrain astrocytes	Lentivirus carrying rtTA	16.48 ±8.6	Dopaminergic (100%)
		In vivo	Ipsilateral striatum, transgenic (GFAP-tTA)110Pop/J mice (adult, P60–P180)	Tet-regulated lentivirus/stereotaxic injection	14.63 ± 8.5 cells/section	Dopaminergic
Small molecules	SLDC * [36]	In vitro	Human cortical astrocytes	Direct application to culture medium	71	Glutamatergic (78%), GABAergic (2%), dopaminergic (1%)
	DFICBY [14]	In vitro	TauEGFP reporter murine astrocytes	Direct application to FCBG* culture medium	89.2 ± 1.4 (TuJ1+, 16 dpi) 77.8 ± 11.1 (NeuN+, 30 dpi)	Glutamatergic, GABAergic
		In vivo	Striatum, mGfap-Cre/Rosa26-tdTomato/TauEGFP mice (P56)	Osmotic minipump for 2 weeks at a constant rate	>350 NeuN/tdTomato+ cells (8 wpi)/127 ± 24 tdTomato+/NEUN+ cells per slice at injection core	Data not provided
	MCMs * [37]	In vitro	Human cortical astrocytes	Applied to culture medium in step-wise manner	68.7 ± 4.2	Glutamatergic (88.3 ± 4%), GABAergic (8.2 ± 1.5%)

* NeAL218: NeuroD1, Ascl1, Lmx1A, and miR218; * SLDC: SB431542, LDN193189, DAPT, and CHIR99021; * DFICBY: DBcAMP, Forskolin, ISX9, CHIR99021, I-BET151, and Y-2763; * MCMs: LDN193189, SB431542, TTNPB, thiazovivin, CHIR99021, VPA, DAPT, SAG, and purmorphamine; * FCBG maturation culture medium: Forskolin, CHIR99021, brain-derived neurotrophic factor (BDNF), and glial cell-line-derived neurotrophic factor (GDNF). Notes: (1) Direct AtN (astrocyte to neuron) conversion is defined as manipulating (forced expression or suppression) 1–2 transcription factors to convert astrocytes directly into induced neurons (iNs), presumably without passing through a multipotent or pluripotent state. Ectopic expression of lineage-specific pioneer transcription factors was postulated to initiate chromatin remodeling which downregulated astrocytic gene expression and upregulated neuron-specific gene expression patterns. (2) Direct AtN reprogramming is conceptualized as transfecting target cells with 3 or more transcription factors, and alternatively, small molecules or signaling pathway modulators that affect multiple epigenetic and/or genetic elements to change astrocytes into iNs, conceivably via a more plastic and potentially multipotent intermediate state. (3) The reported conversion or reprogramming efficiency (%), iN phenotype, and the proportion (%) of each neuronal subtype generated are included (M: male; F: female; for all other abbreviations and information on iN biomarkers, see Supplementary Materials, Tables S1 and S2). (4) Considering the primary focus of this review, some items listed in Table 1 are not further discussed in the text.

### 2.2. Gene Delivery Vehicles

#### 2.2.1. Viral Vectors

Of all the reported vehicles via which TF(s) could be introduced into the astrocyte, AAV appears to be the vector of choice for in vivo cell conversion or reprogramming in adult animals due to a feature of AAV vectors whereby their genomes may persist within cells as episomes in certain conditions [21]. AAV has been deemed safe with low immunogenicity after passing clinical trials and gaining FDA approval [38]. The relatively small particle size of AAV allows it to be delivered in higher titers to achieve greater expression levels of the packaged TF genes. Since certain AAV serotypes can cross the blood–brain barrier (BBB), intravenous (i.v.) administration has been increasingly applied [39]. Critically, AAV is capable of infecting both proliferating (e.g., reactive astrocytes, oligodendrocyte progenitor cells/OPCs, and newly activated GFAP+ NSCs) and quiescent cells (e.g., neurons). If analyzed insufficiently or incorrectly, AAV-mislabeled host endogenous neurons can be wrongly interpreted as iNs and cause misleading conclusions [40]. In fact, some AAV-based studies reported relatively high induction rates [27]. For instance, about 80% of astrocytes infected by AAV-Ngn2/Nurr1 were reported to have been converted into NeuN+ iNs in a murine cortical injury model [41]. Thus, the field may benefit from carefully qualifying the selectivity, dosage, and transfection efficiency of the vectors to minimize cross contamination. Moreover, new technology is needed to differentially determine endogenous mature neurons from possible iNs.

Retroviruses are another commonly used gene vector, especially for in vitro investigations. The main characteristic of retroviruses is that they, when equipped with the wild-type envelopes, mostly infect proliferating cells. Thereby, they spare mature neurons in the host from being transfected, supposedly circumventing the contamination issue associated with AAV. It should be mentioned that under pseudotyping (i.e., engineered with a pre-selected envelope protein that binds a specific receptor in the host cell), retroviruses can infect quiescent cells such as neurons. Because retroviruses may improve the precision and stable expression of the genetic elements being introduced into the genome of the starting cell, they were used to demonstrate that the new neurons/iNs could be derived from dividing glial cells (compared to AAV-*NeuroD1*-induced neuronal conversion of the lineage-traced astrocytes) [40]. Regarding unfavorable characteristics, the size of the transgene is limited to 8–9 kb because of packaging limitations of the retroviral particle [22]. Furthermore, by only infecting dividing cells, retroviruses have a restricted time window during which they can be administered after injury as post-lesion glial proliferation is a transient event (e.g., reactive astrocytes and OPCs; note: NSCs share the astrocytic origin and are GFAP^+^) [22,42]. Retroviruses have mediated varying degrees of AtN induction efficiency. For example, Grestia and colleagues (2019) reported a conversion rate of just 0.35% following treatment with retrovirus-*Neurog2* in a rodent model of focal cerebral ischemia [43]. It has been suggested that the low rate was caused by the augmented number and activity of phagocytotic cells in response to ischemia-increased neuronal apoptosis; these increases engulfed more retroviral vectors hereby preventing the delivery of TFs to the starting cell. In addition, the majority of studies reporting low conversion rate with retroviruses used a single TF, to which the relatively poor outcome was attributed [24].

Lentivirus is a genus of retroviruses capable of establishing sustained and stable gene expression in vitro and in vivo (e.g., the simian immunodeficiency virus (SIV) and the human immunodeficiency virus: HIV). Post-modification lentiviral vectors are considered safe for gene therapies and have been reported to achieve relatively high transfection efficiencies in both normal and diseased conditions in AtN research models. Conversely, similarly to AAV vectors in various settings (e.g., at non-homologous sites where DNA damage may have taken place or by homologous recombination), lentiviruses integrate the transgene into the host’s genome and infect both proliferating and non-proliferating cells (by passing through the nuclear pore complex). This means that they can encounter the same issues of the AAV vectors (see above). One tactic to overcome this problem is to engineer lentiviral vectors to carry the reverse Tet-transactivator (rtTA). In theory, the rtTA-lentiviruses should permit inducible and reversible expression of the transgenes only in the presence of tetracycline (Tet), thereby providing a spatially and temporally controlled gene expression system (for representative AtN studies and references, see Table 1 and Table 2). Still, the reliability of this approach has been hindered by its leaky target gene expression (i.e., the activation of transcription in the absence of Tet).

In earlier research studies, the administration of viral vectors that required their integration into the host’s genome raised safety concerns about insertional mutagenesis, the potential of the viruses regaining reproductive capability, and genotoxic events. Since then, the clinical translatability of AAV-mediated gene transduction has been improved because of the increased recognition that in certain quiescent adult somatic cells (e.g., myocytes and neurons), episomal AAV transduction vectors (replication incompetent) produce stable transgene expressions without changing the genome in host cells [44]. Based on the aforementioned profiles of AAV and retroviruses, it has been proposed that any future AAV-based data should be verified using retroviral vectors that do not transduce neurons to mitigate mislabeling of endogenous neurons [40]. Thereby, systematic verification of AAV- or retrovirus-induced AtN in vitro in well-established non-proliferating astrocytic versus immortalized astrocytic cell lines may offer a more effective approach.

#### 2.2.2. Other Vectors

To overcome the problems resulting from the integration of foreign genes into the host’s genome, non-integrative vectors have been developed, although these vectors typically have a lower gene delivery efficiency than integrative viral delivery systems [45,46]. To this end, the Sendai Virus (SeV) has been identified as a valuable candidate because it is a single-stranded RNA virus. SeV replicates in the cytoplasm, which is non-integrative [47]. Additionally, because it replicates independently of the cell cycle, it is able to generate large copy numbers of the desired transgene. SeV vectors were used to express the Yamanaka factors (i.e., Oct3/4, Sox2, Klf4, and c-Myc) in human fibroblasts and blood cells to produce iPSCs in vitro [48,49,50] and have more recently been used to reprogram porcine fibroblasts into induced NSCs (iNSCs) without passing through an intermediate pluripotent state [51]. Additionally, SeV expressing Gata4, Mef2c, and Tbx5 (GMT) has been used to directly reprogram mouse fibroblasts into induced cardiomyocytes (iCMs) in vitro [52]. It was reported that the SeV-GMT vector achieved a higher (~100-fold) reprogramming rate and quicker (i.e., 10 days) induction of beating iCMs compared with the retroviral (pmx)-GMT vector (30 days) [52]. In that study, the criteria used to define an iCM was restricted to cardiac markers (e.g., α-actinin, cTnT, and αMHC-GFP), morphological analysis (e.g., sarcomeric structures), and electrophysiological recordings. Without examining transcriptomic and epigenetic data, the evidence was not adequate to validate that the induced cells are of a mature cardiomyocyte phenotype. When the SeV-GMT protocol was applied to an in vivo mouse model of myocardial infarction (MI), the intervention was reported to improve ventricular function and MI-induced fibrosis by suppressing collagen I in the infarct border zone [52]. Yet no results were provided to confirm that the iCMs were specifically responsible for the observed functional improvement, nor that they directly or indirectly repressed collagen I. The authors of this paper acknowledged that the mechanisms by which the iCMs mediated the therapeutic effects should be further elucidated by first utilizing in vitro studies. If SeV-formulated protocols are investigated for AtN inductions, study designs should address potential pitfalls based on what has been learned from iCM assays.

Non-viral vectors have also been investigated as non-integrative gene delivery vehicles due to their low immunogenicity and cytotoxicity [53]. Poly(β-amino esters) (PBAEs) are cationic, biodegradable polymers capable of forming complexes (referred to as polyplexes) with negatively charged nucleic acids [54], which can be condensed into nanoscale particles for cellular internalization due to the polymer’s positive charge. The polyplexes are primarily internalized via caveolae-mediated endocytosis and have been engineered to facilitate endosomal release once inside the cell, which subsequently discharges the genetic cargo [55]. While the improved safety of non-viral vectors is considered favorable, their relatively low efficiency remains a major hurdle to broader application in gene transfections compared with viral vector-mediated genetic material transductions. This weakness has also been observed in direct cell reprogramming attempts. It was reported that 5 doses of a PBAE-BAM complex of TFs (Brn2, Ascl1, and Myt1) were required to make mouse embryonic fibroblasts express selected markers of iNs in vitro at a rate of ~8% [56]. A potential way of tackling this issue is to use the topographical cues (microscale and nanoscale patterns that convey information on the three-dimensional (3D) landscape to influence cell behavior) which are thought to prime developing cells for lineage switch [57]. It was shown that topographical patterns helped to reduce the number of doses required to achieve substantial levels of cell phenotype change with non-viral vectors, since one dose of PBAE-BAM polyplex on hierarchical patterns achieved a reprogramming rate in murine embryonic fibroblasts equivalent to five doses of PBAE-BAM in a regular control setting [57].

Importantly, it seemed that polymer selection is also critical to achieving adequate transfection efficiencies in direct AtN conversions. For example, out of 5 selected polymers, a polyplex formed of polymer 536 (60 *w*/*w* + 1 µgCm^−2^ DNA) and SOX2 (PBAE-SOX2) was found to yield the highest transfection rate in primary human astrocytes in vitro (i.e., 43.2 ± 5.0%), which was higher than that of the commercially available transfection reagent Lipofectamine™ 2000 [58]. Although this approach was reported to convert the human astrocytes into Nestin and Tuj1-expressing neuroblasts, it is not possible to conclude what percentage of the transfected cells were converted because quantitative data were not provided. In addition, SOX2-mediated AtN induction required supplemental treatments such as valproic acid and Noggin to generate iNs [59]. Hence, caution should be introduced regarding the risk of tumorigenesis carried by Noggin due to its role as a bone morphogenetic protein antagonist.

### 2.3. PTBP1 Knockdown

**Figure 2:**
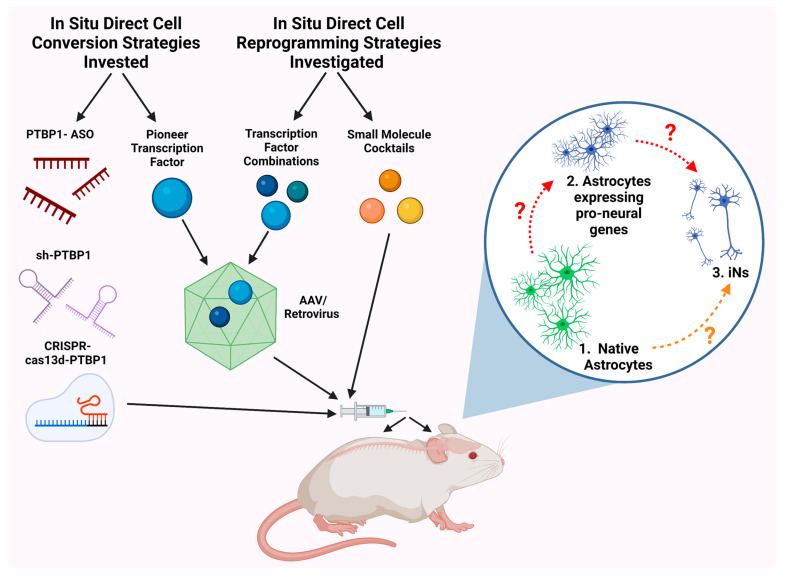
Schematic illustration of the general principles used to guide cell conversion or reprogramming for attaining neurons from astrocytes in adult animals. Common AtN conversion strategies were based on the manipulation of a single lineage-specific transcription factor (TF) to alter astrocytic chromatin accessibility and drive neuronal gene expression patterns in astrocytes. This was experimentally operated via two mechanisms: (1) the ectopic expression of a pioneer factor packaged into a viral vector; (2) the genetic knockdown of polypyrimidine tract binding protein 1 (PTBP1) using short-hairpin RNAs (shRNA), antisense oligonucleotides (ASO), or CRISPR-cas13d. Conversely, direct AtN reprogramming strategies utilize 3 or more TFs, small molecules, or signaling pathway modulators affecting multiple epigenetic and genetic elements of the starting astrocytes. For in vivo studies, both approaches relied on microinjection of the AtN induction factors/molecules into the target brain or spinal cord region. While the data and biological underpinnings of all AtN tactics remain to be fully validated/elucidated (indicated by question marks), it was suggested that direct conversion protocols induced a direct transition from one somatic cell type into another without passing through a multipotent or pluripotent state (orange dashed arrow) and direct reprogramming procedures were thought to return the starting cell to a more plastic and potentially multipotent-like state before triggering differentiation into the neuron-like phenotype (red dashed arrow; all images created using BioRender.com, accessed on: 17 November 2022).

Another published approach to achieving AtN induction was the knockdown of the polypyrimidine tract binding protein 1 (PTBP1) by applying short hairpin RNAs (shRNA), antisense oligonucleotides (ASO), or CRISPR-cas13d (Figure 2) [33,34,35,60]. The downregulation of PTBP1 seemed to promote a neuronal phenotype by interfering with PTB-dependent alternative splicing and the miR circuits that gatekeep the RE1 (repressor element-1) silencing transcription factor (REST) complex. In non-neuronal cell types, PTBP1 mRNA encodes a suppressor of alternative splicing which maintains the non-neuronal phenotype (e.g., fibroblasts); however, in neuronal differentiation, miR-124 targets PTBP1 mRNA and represses its expression, leading to an accumulation of PTBP2 which drives neuron-specific alternative splicing and neuronal differentiation. Therefore, it was reasoned that knocking down PTBP1 in non-neuronal cells might mimic the actions of miR-124 (expressed in neurons but not astrocytes) to artificially drive neuron-specific alternative splicing patterns for altering the cellular proteome. Xue et al. (2013) presented data that the downregulation of PTBP1 promoted fibroblast transdifferentiation into iNs [61].

This approach has since been reported to generate dopaminergic iNs from astrocytes that integrated into the existing striatal network to reduce motor deficits in a rodent 6-OHDA-lesion model of Parkinson’s disease [34]. The data suggested that the newly induced dopaminergic iNs were capable of stimulation-dependent dopamine release in vivo, which was shown through a chemogenetic arrangement called “Designer Receptors Exclusively Activated by Designer Drugs” (DREADD) to assess if the iNs were directly responsible for the observed behavioral improvements. For the latter intervention, mice were treated with an AAVsh-PTBP1 vector in which the RFP was replaced with a flox-embedded inhibitory variant of the human muscarinic receptor (hM4Di) at the time of injury, which enabled iNs to initiate cell membrane hyperpolarization. When the AAVsh-PBTP1-DREADD construct-treated mice were administered clozapine-N-oxide (a DREADD agonist/designer drug), their Parkinsonian symptoms were improved within 40 min. In contrast, in animals lacking the hM4Di receptor, Clozapine-N-oxide exerted no effect. However, this conclusion assumed that the AAV vector was entirely selective for its intended target, which, as seen from previous studies, is unlikely. In addition, dopamine has an elimination half-life of 2–5 min in rodents. It is not clear why a latency of about 40 min was required before the dopamine-driven behavioral improvement was observed. In general, for the TF-mediated AtN product, the field has yet to validate how long the iN phenotypic characteristics last and to uncover the mechanisms that enable iNs (i.e., previous astrocytes) to be functionally connected with other host neurons in a non-disruptive, non-impeding, and coherent manner.

### 2.4. Small Molecules

Astrocytes were shown as being directly reprogrammed into iNs by small molecules (≤1000 Daltons). The reported regimens seemed to be capable of diminishing expressions of astrocyte-specific genes/markers (e.g., S100 and GFAP) and upregulating endogenous neurogenic genes, circumventing the need to introduce exogenous TF genes [62]. This approach has shown several distinct properties, including the benefit that they do not integrate into the genome of the starting cell and therefore curtail the risk of activating neoplastic genes to trigger tumorigenesis. Additionally, they appeared to be non-immunogenic and their effects were reversible if the molecules were removed too soon. This feature does require prolonged exposure to the formulated molecules for the induced phenotype to be sustainable in certain timeframes [63]. Numerous investigations have demonstrated that exposing astrocytes to varied small molecule cocktails initiated the activation of neurogenic gene expression and the transcription of endogenous proneural factors including NeuroD1 and Neurog2 [14,37,62]. A treatment comprised of 4 molecules (CHIR99021, DAPT, LDN193189, and SB431542) suppressed glial cell-specific genes (e.g., *FN1* and *MYL9*) within 24 h following the administration and modulated the activity of several signaling cascades including the upregulation of Notch and downregulation of JAK/STAT signaling pathways [63]. The five molecules in the optimized FICBY cocktail (i.e., Forskolin, ISX9, CHIR99021, I-BET151, and Y-27632) were demonstrated to work synergistically in vivo to reprogram astrocytes into functioning iNs in the adult mouse brain [14]. The criteria used to define an iN in this study included the mature neuronal marker NeuN, striatum-specific subtypes of genes (e.g., *Gad1*), cortex-specific neuronal markers (e.g., CTIP2), synaptic activity genes (e.g., *Bsn*), conditional lineage tracing or transsynaptic tracing, and whole-cell patch clamp recordings of action potentials and inward/outward currents from iNs in the striatum and cortex. These outcomes provided markers and certain operational features of a neuron-like cell. Future studies need to show specific epigenetic, genetic, and neurological changes in their time courses following the cocktail treatment.

In the absence of either ISX9, I-BET151, CHIR99021, or Forskolin, the FICBY cocktail failed to generate any NeuN+/tdTomato+ cells in cell culture or the striatum of the mouse model (8 weeks post injury: wpi), and the absence of Y-27632 markedly diminished reprogramming efficiency [14]. The data suggested that the synergistic interaction between these molecules was crucial for successful reprogramming. However, information is needed to rule out the possibility that the result was a confounding variable. Froskolin, which is a cell-permeable activator of adenylyl cyclase, appeared to be particularly important because when given at higher doses (300 µM, released at a rate of 0.25 µL/h for 14 days via osmotic minipump) it generated more mature iNs in vivo as assessed by the expression of NeuN, a mature neuronal marker, and electrophysiological properties [14,63]. No additional treatment was given to sustain the iN phenotype upon cessation of small molecule exposure in that study; the iNs seemed to remain NeuN^+^ and display electrophysiological characteristics resembling those of functioning neurons at 8 weeks ex vivo. However, possible mechanisms underlying this phenotypic sustainability were not explored. This weakness must be addressed since somatic cells are generally strong in maintaining their genetic integrity.

Methods have nevertheless been under development to help stabilize the induced phenotype after the removal of small molecules, represented by strategies to inhibit specific cell de-differentiation signaling pathways and to apply biomaterials containing an organ-specific decellularized extracellular matrix (dECM). For example, Notch signaling has been implicated in the de-differentiation of medulla neurons to neuroblasts in Drosophila melanogaster in vitro, and suppression of Notch signaling with the zinc finger TF Nerfin-1 exhibited effectiveness in preventing the de-differentiation process [64]. Additionally, scaffolds containing brain dECM were tested to preserve the native neural tissue microenvironment by supplying interstitial growth factors, collagens I and II, laminin, and cytokines important to restore the balance of differentiation and de-differentiation cues. Because the expression of ECM proteins is brain-region specific [65,66], dECM from the striatum was thought to help in establishing and maintaining a dopaminergic neuronal phenotype. This strategy was used on a hydrogel-based chip or in 3D culture models to recapitulate the in vivo brain environment [67]. Nonetheless, how specific and sustainable these tactics can exert the anticipated effects has yet to be investigated.

### 2.5. Other Tactics

#### 2.5.1. Micro-RNAs (miRs)

It was uncovered that as NSCs exited mitosis to initiate neural lineage differentiation, they underwent an ATP-dependent chromatin remodeling switch that was essential for the development of post-mitotic neurons [68]. The data suggested that miR-9*, the counter-strand of miR-9 and miR-124 (miR-9*-124: miR-9-3′ prime and miR-124-5′ prime), drove this transition by repressing BAF53a (also known as Actin-like protein 6A: ACTL6A), which facilitated the exchange of neural progenitor BAF sub-units (BAF53a and BAF45a) for neuron-specific BAF subunits (BAF53b and BAF45b) [68]. This finding paved the way for exploring a new experimental protocol in which miR-9* and miR-124 were administered for direct neuronal conversion. miRs have also been enrolled as mediators in the field of direct AtN inductions. In theory, miRs are able to modulate gene expression at the post-transcriptional level, and their small sizes allow more efficient delivery into cells under proper conditions. When expressed in conjunction with TFs known to promote a motor neuron phenotype (e.g., ISL1 and LHX3), miR-9*-124 were reported to reconfigure chromatin accessibility at pro-neural genetic loci and trigger DNA methylation, which promoted the conversion of adult human fibroblasts to “induced motor neurons” (iMNs) in vitro [69].

In that report, however, the criteria for concluding that the iNs were of a MN phenotype were limited to the expression of MNX1 (a marker of MNs) and the detection of cytoplasmic CHAT, the rate-limiting enzyme of acetylcholine synthesis, and SMI-32, a neurofilament protein commonly found in MNs. More recently, miR-9*-124 alone was found to be sufficient to convert human fibroblasts to neurons in vitro [70] by erasing the fibroblast network and activating a neuronal gene expression pattern (also see above) [71]. miR-9*-124 was shown to erase the fibroblast network by directly targeting and repressing Krüppel-like factors (KLFs) 4 and 5, before activating the downstream molecule RN7SK (RNA component of 7SK nuclear ribonucleoprotein), which induced a gene expression pattern that drove the cells towards a neuronal fate [71]. Church et al. (2021) began to establish different sets of in vitro miR-based protocols to generate specific subtypes of human iNs from fibroblasts, including striatal medium spiny neurons as well as MNs of the spinal cord and cerebral cortex [72]. It is important to point out that whether these methods can be utilized for in vivo applications has yet not been determined. Furthermore, since fibroblasts are highly plastic, it is unclear how effective this approach may be in differentiated somatic cells such as astrocytes, especially when they are in pathological conditions such as cytotoxic edema after neurotrauma [73].

#### 2.5.2. DNA Binding Domains

Transcription factors are modular in nature and have been classified based on the structure of the domain that binds DNA. One type of DNA binding domain required zinc and has been defined as a zinc finger motif. In this group, the C2H2 zinc finger proteins (ZfP) represent one of the most common types of DNA binding domains. ZfP are small structural motifs in which one or more zinc ions are coordinated to stabilize the folds into a stable three-dimensional assembly [74]. In the C2H2 class of zinc finger transcription factors, a variety of extended sequence motifs exist. They regulate subcellular localization, DNA binding, and gene expression by controlling the selective association of TFs with each other or with other cellular components. For the C2H2 class of zinc fingers, the associated modules are the poxvirus and zinc finger (POZ) domain, also termed as the BTB domain (Broad Complex, Tramtrack, and Bric-a-brac), the Krüppel-associated box (KRAB), and the SCAN domain (named after SRE-ZBP, cTfin51, AW-1, and Number 18 cDNA; also known as the leucine-rich region: LeR) [75].

C2H2-zinc finger proteins are abundantly expressed in the developing brain to modulate early CNS patterning, control NSC activities, and regulate their exit from pluripotency [76]. CRISPR-mediated knockout of ZrP217 and ZfP516 in embryonic stem cells (ESCs) prevented their exit from pluripotency and inhibited neuronal differentiation [77]. Hence, DNA-binding domains are emerging as another tool in the cell reprogramming toolbox. Engineered ZfPs can be genetically fused to transcriptional activators (e.g., VP16 acidic trans-activator monomer) or repressors with their DNA binding domains precisely directed to the target gene [78]. The Krüppel-like Zfp521 was shown to activate early neural genes (e.g., *Pax6*, *Sox1*, and *Sox3*) and has been deemed sufficient to directly convert both fetal fibroblasts and mature astrocytes into iNs in vitro. Zarei-Kheirabadi and colleagues (2019) found that in cultured astrocytes, Zfp521 achieved a higher AtN rate than *Sox2* and transformed astrocytes to iNs (both glutamatergic and GABAergic) likely through impacting multiple factors related to multipotent NSC states [79]. However, the process was relatively slow compared with other single factor-induced AtN: it took 4 weeks for the mature neuronal markers to become detectable, compared with 1 week in NeuroD1-mediated AtN conversion in a murine in vivo ischemic stroke model [20]. When applied in vivo to a rat T9-11 SCI model, Zfp5210-expressing lentiviral vectors converted endogenous astrocytes into iNs, which was accompanied by an improvement in SCI-caused hindlimb locomotion deficits relative to controls. Furthermore, the presence of GAP-43 (growth-associated protein 43), a marker of axon growth cones, and the reappearance of motor-evoked potentials were also presented. Contrariwise, no explanation was given regarding why T9-11 iNs were related to hindlimb locomotion improvement and how iNs could give rise to or induce axon regeneration and/or induce re-exhibition of evoked potentials; these issues together with that of using solely neuronal markers (e.g., MAP2) to define iNs suggested that more work is needed before a more concrete conclusion can be drawn (see Section 3.4).

#### 2.5.3. CRISPR

The clustered regularly inter-spaced short palindromic repeat (CRISPR) technology is a more recent addition to the direct cell reprogramming methodology repertoire. Originally, the technology comprised of an RNA-dependent DNA endonuclease (Cas9) and a small synthetic guide RNA (sgRNA) which facilitated targeted double-stranded DNA breaks at specific genetic loci. Since then, a deadCas9 (dCas9) mutant has been developed that is devoid of nucleolytic activity but can still perform targeted DNA binding at specific loci including promoters and enhancers [80,81]. This tactic has been exploited to deliver activating or repressive cargo to the target gene, which permits precise alterations of genetic expression via epigenetic regulation [82]. The main advantage of the dCas9 tactic compared with traditional cell reprogramming or conversion methods is the ability to simultaneously modulate the expression of multiple genes by tiling a variety of different sgRNAs, hereby enrolling several dCas9 proteins [82]. Epigenetic effector domains such as TET-1 and p300 have also been fused to dCas9 as a method of selectively removing the epigenetic barriers (e.g., CpG island methylation and H3K27 acetylation) known to prohibit cell fate reprogramming [80].

When verified 1 week later in the SunTag-p65-HSF1 (SPH)-transgenic mouse model, the administration of dCas9 and AAV-sgRNAs (5 × 10^12^ gc/mL) was reported to have reprogrammed the mCherry-labeled midbrain astrocytes into functional NeuN+ iNs through activating Ascl1, NeuroD1, and Neurog2. In this study, the functionality assessment was restricted to whole-cell patch clamp recordings of neuronal electrophysiological characteristics. Repeating this study with the use of a retroviral delivery method, wild-type animals as control groups, and a more rigorous analysis of the iN phenotype would likely be informative to further substantiate these data. More recently, the CRISPRcas13d (CasRx) RNA-targeting system was utilized to mediate *Ptbp1* knockdown and the subsequent conversion of striatal astrocytes into iNs with dopaminergic features in a 6-OHDA mouse model of Parkinson’s disease [35]. CasRx, the smallest of the Cas proteins, displayed high target specificity. It was therefore taken as a candidate for in vivo applications. Based on the co-expression of mCherry and tyrosine hydroxylase (TH; the rate-limiting enzyme of dopamine synthesis), the conversion rate was 19 ± 0.4% (*n* = 5) at 1 month; three months post injection, 32 ± 7% (*n* = 3) of mCherry^+^ cells expressed the dopamine transporter (DAT), a marker of mature dopaminergic neurons. The study attained the conversion rate data from relatively limited samples of mice and did not use statistical methods to pre-determine sample sizes [35].

## 3. Major Challenges in the Field of Direct AtN Reprogramming and Conversion Research

### 3.1. Transcriptional Mechanisms and Quality Control

Ideally, an iN should reliably recapitulate major genetic, epigenetic, biochemical, and functional characteristics of endogenous adult neurons in individual and networking manners. Yet currently, reprogrammed/converted cells are being characterized as iNs mostly based on rtPCR and immunoreactivity evidence of neuronal genes/markers (e.g., NeuN), as well as patch clamp recordings to show the cell membrane’s electrophysiological excitability (Table 2). Evidently, these criteria are not sufficient for defining functional neurons in vivo. For neuronal phenotype validation, the advent of single-cell omics technologies has enabled a deeper understanding of the genomic, transcriptomic, epigenomic, proteomic, and metabolomic signatures that underlie an adult neuron. These technologies have landed the means with which to measure finely clustered distinctions between different cell types. They have also provided valuable possibilities to investigate the biological and regulatory processes that govern cell phenotypic presentations and stress-induced molecular manifestations following cell conversion or reprogramming manipulations [83]. Armed with this information, a more stringent set of molecular criteria for what differentiates an iN from a “neuron-like cell” or a physiological adult neuron may be eventually devised, which in turn should be utilized as one of the quality control measures. To move toward a qualitatively higher level, the field may need to first establish specifically stratified standards to individually validate each neuronal subtype. This approach can enable more tangible comparisons between studies about the neurobiological profile of iNs. Furthermore, all of the “reprogrammed or converted neuron-like cells” must be able to interconnect with a set of host neurons, astrocytes, and oligodendrocytes (i.e., a neural/neuronal circuit) that jointly subserve a specific physiological function in vivo before they can be defined as iNs.

To this end, a research investigation has presented some pilot data about the hierarchical process of the reprogramming of highly plastic fibroblasts to cells with neuronal markers through applying a BAM (*Brn2*, *Ascl1*, and *Myt1l*) protocol [84]. In recent years, more insight has been gained regarding how the representative TFs and small molecules altered not only the transcriptional but also the genetic, epigenetic, and metabolic landscapes [63,85,86,87]. In the early stages of the reprogramming or conversion process, over 1500 differentially expressed genes were identified; in particular, chip-sequencing analysis suggested the genome-wide direct binding sites of *Ascl1*, which included the regulatory regions of *klf10*, *Myt1*, and *Neurod4*, with *klf10* being involved in neuritogenesis of the induced cells, *Myt1* essential for the electrophysiological maturation, and *Neurod4* affecting conversion efficiency [86].

Several studies have also begun to map out a time course of key events, including the activation of signaling pathways and the different waves of gene expression triggered by the TF or small molecule application [63,87]. For example, during the AtN induction of human cortical astrocytes (HA1800) to iNs in vitro, the expression of *NeuroD1* itself was downregulated 3 days post-induction (dpi), accompanied by an upregulation of a few genes including *NeuroD2* and *NeuroD6*. This indicates that at 3 dpi, NeuroD1 relayed its effects onto downstream secondary factors such as other NeuroD family members that were probably transcriptional activators to mediate neuronal differentiation [87].

**Table 2:** Representative In Vitro and In Vivo Direct AtN Conversion and Reprogramming Studies.

Induction Factor(s)	Vector/ Delivery System	Cell Type/ Anatomical Target	Induction Efficiency (%)	Criteria	iN Phenotype/ Criteria		iN Features
*In vitro astrocyte to neuron reprogramming*							
NeAL218 + MP * [26]	Lentivirus carrying rtTA [26]	ATCC (SVGp12, cat. n CRL86-21), midbrain (hIAs)	30.97 ± 5.3	TH+, MAP2+ (84.6 ± 1.9%), TUBB3+	Dopaminergic (100% of iN)—DDC, SLC6A3, FOXA2, EN1, and SLC18A		Simple neuron-like morphologies and lack emDAs membrane properties
NeAL218 + RTMP * [26]		ATCC (SVGp12, cat. n CRL86-21), midbrain (hIAs)	16.48 ± 8.6	TH+, TUBB3+, MAP2+, SYN+	Dopaminergic (100% of iN)—DDC, SLC6A3, ALDH1A1, and KCNJ6		Ca^2+^ response upon depolarization (55 mM KCl), generate AP, sEA + AP at 13–17 days, current clamp recordings show different firing properties upon current injection (none, single AP, multiple AP), and 2/7 (≈29%) generate multiple AP
		Lonza (normal human astrocytes, cat. n CC-2565), hPAs	12.4 ± 2.7	TH+, MAP2+, RBFOX3+	Dopaminergic (100% of iN)—DDC, SLC6A3, ALDH1A1, KCNJ6, and PBX1		
*In vitro astrocyte to neuron conversion*							
Ascl1 (Mash1) [29,88]	Lentivirus FUGW [29]	Isolated from P5–P7 mice, postnatal dorsal midbrain	76.8 ± 6.4	Tuj1+ MAP2+, and Synapsin I+	Glutamatergic (19.4%)—blocked by CNQX, GABAergic (8/38, ≈21%)—blocked by Bicuculline		Produce AP and sPSC in 85.3%
	Retroviral VSV-G [88]	C57BL/6 mice P5–P7, pNCC	37 ± 11% and 14 ± 2% Tuj1+, and >40% TuJ1-	TuJ1+	No TuJ1+/Tbr1+, no clear nuclear staining for Ascl1 (Mash1)		Intrinsic excitability, generate typical neuronal AP, and virtual absence of spontaneous synaptic input
Neurog2 (Ngn2) [88,89]	Retroviral VSV-G [88]	C57BL/6 mice P5–P7, pNCC	>85%, 71 ± 16%, and 16 ± 18% clones TuJ1+, and ~10% clones TuJ1-	TuJ1+	Glutamatergic (≈33%)—TuJ1+/Tbr1+, blocked by CNQX GABA (polysynaptic, UD)— >5 ms delay, blocked by both CNQX and Bicuculline		Fire repetitive AP, ↑ negative resting mV, ↓ IR, ↑ AP amp over time, functional but ↓ PS response, and not generate SR from neighboring neurons
	pCAG-IRES-DsRed (self- silencing, long-acting) [89]	C57BL/6J or GLAST::CreERT2/Z/EG mice P5–P7, pNCC	70.2 ± 6.3%,	BIII tubulin+, GFAP-	Glutamatergic (58.3%)—BIII tubulin+/vGlut1+ puncta (85.4 ± 5.0%) GABA (0%)		MAP2 in 2–3 weeks and Ca^2+^ transients (63.8%)
In neurosphere [89]			91.4 ± 2.2%	MAP2+	Glutamatergic—MAP2+/vGlut1+, AC/SC (9/21, ≈43%), CNQX-sensitive sSC (8/30, ≈27%)		Low IR
Dlx2 [89]	pCAG-IRES-DsRed (self- silencing, long-acting) [89]	C57BL/6J or GLAST::CreERT2/Z/EG mice P5–P7, pNCC	35.9 ± 13.0%	BIII tubulin+, MAP2+	GABAergic—Autapses, vGlut1-, BIII tubulin/vGaT+ (33.7 ± 3.6%), sSC with slow decay time (9/33, ≈27%), AR blocked by Bicuculline		Neuron morphology, fire AP, distinct firing patterns (regular, stuttering, and low-threshold), 7/9 (≈78%) immature firing pattern, and 2/9 (≈22%) mature interneuron-like firing pattern
In neurosphere [89]			94.7 ± 0.3%	MAP2+	GABAergic—MAP2+/vGaT+ puncta, slow decay time (9/10, ≈90%, UD)		↓ IR and no Ca^2+^ transients
**Induction Factor(s)**	**Vector/** **Delivery System**	**Animal Model/Sex**	**Anatomical** **Target**	**Direct Reprogramming** **Efficiency (%)**	**Criteria**	**iN Phenotype/** **Criteria**	**iN Features**
*In vivo astrocyte to neuron reprogramming*							
ALN * [90]	Cre-inducible AAV5/injection	Adult GFAP-Cre mice (P84–P112)	Striatum	46.8 ± 2.9	NeuN+	Glutamatergic—vGlut1+ (16%) GABAergic—GAD65/67+ (68%)	rMP (−61.4 ± 9.7 mV), AP mean amp (33.5 ± 2.29 mV), and AP threshold (25 ± 7.19 pA)
NeAl218 * [26]	Tet-regulated NeAL218 lentiviruses/stereotactic needle injection	Adult Tg(GFAP-tTA)110Pop/J mice (P60–P180)	Ipsilateral striatum	14.63 ± 8.5	TH+	Dopaminergic—TH+/SLC6A3+, RBFOX3+, NR4A2+, and PBX1+	TH+/SLC6A3+ iNs produced I_h_
*In vivo astrocyte to neuron conversion*							
Ascl1 [29]	AAV/ micropipette injection [29]	Adolescent WT mice (P12–P15), M + F	Dorsal midbrain	93.1 ± 1.7	NeuN+	GABAergic—NeuN+/Gad1+ (13.2 ± 4.2%) Glutamatergic—NeuN+/VGLUT2+ (6.5 ± 2.2%)	Producing AP, sPSC observed, IOC in VCM, MΩ (177.3 ± 16.6), and ↓ RMP (−61.9 ± 1.0)
		Adult WT mice (P60), M + F		92.1 ± 1.5	NeuN+	GABAergic—NeuN+/Gad1+ (11.7 ± 4.0%) Glutamatergic—NeuN+/VGLUT2+ (6.3 ± 1.3%)	Producing AP, sPSC observed, IOC in VCM, MΩ (240.0 ± 81.9), and ↓ RMP (−61.0 ± 1.2)
			Striatum	64.4 ± 3.4	NeuN+	GABAergic (according to electrophysiological test performed)	Fire APs in CCM (13/16, ≈81%), sEPSC and sIPSCs (12/16, ≈75%), and IOC in VCM (15/16, ≈94%)
			Somatosensory cortex	93.9 ± 1.2	NeuN+	Glutamatergic or GABAergic (according to electrophysiological verification)	Record 163.3 ± 35.9 MΩ, dMP (−67± 2.2 mV), APs, IOC, sEPSC, and sIPSCs
	AAV-FLEX/ micropipettes injection [29]	Adult *Aldh1l1*–Cre transgenic mice (P60), M + F	Dorsal midbrain	90.1 ± 2.1	NeuN+	GABAergic (according to electrophysiological verification)	Exhibit firing patterns identical to midbrain endogenous GABAergic neurons
	AAV/needle injection [29]		Injured dorsal midbrain	54.2 ± 6.9	NeuN+	Glutamatergic or GABAergic (according to electrophysiological verification)	424.7 ± 88.7 MΩ, rMP (−61.2 ± 1.6 mV), IOC in VCM, rAPs fired in CCM, sEPSC, and sIPSCs
NeuroD1 [21,22,23]	AAV/stereotactic needle injection and infusion pump [21]	Adult *Macaca mulatta* (9–21 years old), M	Cortex	94.4 ± 5.5	NeuN+/ Tbr1+	Glutamatergic—Tbr1+, projection neurons	↑ SV2 and significantly recovered MAP2
	AAV9/ stereotactic needle injection [23]	Adult WT mice (P90–P180), M + F	Cortex	90.6 ± 5.2	NeuN+	Glutamatergic—vGlutT1+ GABAergic—GAD67+	↑ SMI32, ↑ vGluT1 and GAD67, large Na^+^/K^+^ currents (13/15, ≈87%), rAPs (7/10, ≈70%), glutamatergic SE (10/13, ≈77%), and GABAergic SE (9/13, ≈69%)
	Cre-FLEX AAV/needle injection [22]	Adult WT mice (P60–P120), M + F	Stab-injured dorsal horn T10	~95.0	NeuN+	Glutamatergic—NeuN+/Tlx3+ (62.6 ± 3.3%) GABAergic—NeuN+/Pax2+ (8.8 ± 1.3%)	rAPs, large Na^+^/K^+^ current, robust spontaneous EPSCs, and no difference in Na^+^ current and sEPSCs compared with neighboring native neurons
			Contusive SCI model T10 acute phase	~55.0	NeuN+	Glutamatergic—Neu+/Tlx3+ in dorsal horn	↑ SV2
			Contusive SCI model T11–T12 chronic phase	>95.0	NeuN+	Glutamatergic—Neu+/Tlx3+ in dorsal horn	↑ SV2
Neurog2 [24]	AAV/stereotactic needle injection	Adult WT mice	Dorsal midbrain	96.3 ± 1.7	NeuN+	Glutamatergic—NeuN+/VGLUT2+ (64. 97 ± 8.04%) GABAergic—NeuN+/Gad1+ (2.26 ± 2.07%)	Multiple APs, IOC in VCM, EPSC, MC and the IR of iN are largely comparable with local neurons, and neuronal profile
			Dorsal horn T8–T10	80.11 ± 5.42	NeuN+	Glutamatergic—Tlx3+ (50.9 ± 8.8%) GABAergic—Pax2+ (38.5 ± 8.3%)	Produce IOC in VCM, multiple APs (9/11, ≈82%; ↓ AP amp), and MC and iR comparable to native neurons
	AAV/injection from L1–L2 dorsal surface		Transected SC T8–T10	41.62 ± 22.82	NeuN+	Data not provided	Data not provided
Ptbp1 knockout [35]	AAV-GFAP-CasRx-Ptbp1 with gRNAs 5 + 6 for Ptbp/stereotactic injection	Adult C57BL/6 mice (~P70)	Striatum	48.00 ± 10.00	NeuN+	Glutamatergic—50% iNs glutaminase+	Data not provided
			Ipsilateral striatum/PD model	32.00 ± 7.00	TH+	Dopaminergic—TH+/DAT+ (31 ± 7%), ~15% TH+/DDC+, ~37% TH+/FOXA2+ iNs were ALDH1A1+, GIRK2+, and CB–	rAPs (20/22, ≈91%) in response to depolarizing current injection in the CCM, sPSC observed in VCM (Vc = −70 mV), delayed voltage rectification induced by I_h_ (4/10, 40%), and majority iNs were VMAT2+
NeuroD1 + Dlx2 [22,27]	rAAV2/5/stereotactic bilateral needle injection [27]	Adult WT mice (P60–P140), M + F	Striatum	72.7	NeuN+	MSN—NeuN+/DARPP32+ (55.7%) GABAergic—NeuN+/GAD67+ (83.9%) GABAergic—NeuN+/GABA+ (85.0%) Interneurons—NeuN+/PV+ (9.6%) NeuN+/SST+ or NPY+ or CalR+ (<5%)	Data not provided
		Adult R6/2 transgenic mice (P60–P150), M + F		78.6	NeuN+	MSN, GABAergic, and interneuron; additional expression: DARPP32 (56.6%), GAD67 (82.4%), GABA (88.7%), PV (8.4%), and <5% (SST, NPY, CalR)	iNs rAPs (17/18, ≈94%), 72.2% firing at <80 Hz, 22.2% firing at >80 Hz, detected sEPSCs and sIPSCs in all iN, and ↑ iR, ↓ cC, ↓ RMP, and ↓ AP amp compared with control
		Middle-aged YAC128 transgenic mice (15 months), M + F		50.0	NeuN+	MSN, GABAergic, and interneuron; additional expression: DARPP32 (29.8%), GABA (half), and PV (3.9%)	Data not provided
	Cre-FLEX AAV/needle injection [22]	Adult WT mice (P60–P120), M + F	Stab-injured dorsal horn T11–T12	N/A	Tlx3+ Pax2+	Glutamatergic—Tlx3+ (56.2 ± 3.4%) GABAergic—Pax2+ (32.5 ± 2.1%)	Data not provided
Ascl1 + Nurr1 [41]	FLEX-switch AAV/ microinjection	Adult mGFAP-Cre mice (P60–90), M + F	Injury cortex model	40.0 (24 dpi) 70.0 (72 dpi)	NeuN+	iNs variable morphology	* Ascl alone served as a control and was shown to have a conversation efficiency of ≈20.0% (NeuN+)
Neurog2 + Nurr1 [41]				53.0 (24 dpi) 80.0 (72 dpi)	NeuN+	NeuN+/CUX1+ iNs in upper layer, NeuN+/CUX+ iNs in deeper layer; both displayed stereotypical pyramidal-shaped cell soma; single and combinatorial labeling for CUX1, SATB2, and BRN2+ iNs in upper layers FOXP2+, CTIP2+, TLE4+, and TBR1+ iNs in lower layer	rMP, iR, APs comparable to endogenous neurons, and E/I input blocked by NBQX. * Nurr1 alone served as a control and was shown to have a conversation efficiency of ≈20.0% (NeuN+)

* ALN—Ascl1, Lmx1a, and Nurr1; * NeAl218—NeuroD1, Ascl1, Lmx1a, and miR218 (for abbreviations and iN biomarker information, see Supplementary Materials, Tables S1 and S2). Notes: (1) In vitro astrocyte to neuron (AtN) direct reprogramming using a cocktail of induction factors and specific molecular protocols to produce “induced neurons” (iN). These protocols were reported to unwind DNA so that transcription factors could enter and induce changes in phenotype. (2) In vitro AtN direct conversion was performed by using up to two transcription factors to generate iNs. (3) In vivo AtN direct reprogramming was performed by using ≥3 transcription factors or small molecules affecting multiple epigenetic and genetic elements to reprogram astrocytes into reported glutamatergic, GABAergic, or dopaminergic iNs. (4) In vivo AtN direct conversion used up to two transcription factors to generate reported glutamatergic, GABAergic, or dopaminergic iNs. (5) The delivery system of induction factors, animal sex (M: male; F: female), and anatomical target for each study are exhibited. (6) Reported reprogramming/conversion efficiency (%) is presented based on the specific criteria of each study for defining an iN. (7) Essential information on the functional assessment of iNs is included.

### 3.2. Specific Epigenetic Mechanisms

Non-CpG methylation in high levels is a distinct epigenetic signature of neurons, and thus theoretically should be recapitulated in iNs. Whole-genome DNA sequencing has shown that the ectopic expression of BAM factors in murine fibroblasts could establish global non-CpG methylation patterns in the resulting iNs [91]. The non-CpG methylation patterns were reported to resemble those found in cortical neurons. However, the paper did not contain data to define which subtypes of neurons the iNs became. It is conceivable that differences in non-CpG methylation patterns exist between neuronal subtypes. Moreover, this epigenetic trait should be verified in iNs derived from other AtN induction protocols (Table 2) before it can be further validated.

Researchers have found that the trimethylation of histone 3 at lysine 4 (H3K4me3) is a mark of transcriptionally active genes [92]. What appeared to occur during direct cell reprogramming and conversion was a gradual loss of H3K4me3 from the promoters of starting cell-specific genes and an accumulation of these marks at the promoters of target cell-specific genes [93]. It was shown that the knockdown of *KMT2B* (coding for lysine-specific histone methyltransferase 2B that produces H3K4me3) in fibroblasts substantially reduced a BAM-protocol-mediated iN reprogramming rate and produced more cardiomyocyte-like cells instead [94]. The reduction in reprogramming efficiency was attributed to downregulation of key iN maturation genes (*Zfp612*, *Lass4*, and *Arnt2*) which were usually upregulated early in the reprogramming process, and dysregulation of the repressor ZFP238, a primary target of Ascl1. Therefore, the ablation of *KMT2B* prevents BAM factors from interacting with their appropriate counterparts. The increased production of cardiomyocyte-like cells was thought to also result from the loss of *KMT2B* to prevent the silencing of alternative fates, allowing the fibroblasts to reroute down a different (i.e., cardiomyocyte) differentiation pathway. However, the mechanism by which this occurred was not investigated. It would be illuminating to investigate whether the same phenomenon existed in AtN processes.

Other histone modifications reported to influence cell fate and maturation during direct reprogramming or conversion include H3K27me3 and H3K27ac (or H3K4me3), which serve as markers of transcriptional repression and activation, respectively [93,95]. Neuronal conversion with *Ascl1* also augmented the expression of *Dnmt3A* (the protein-coding gene for DNA methyltransferase 3α) which promoted de novo methylation at the promoters of fibroblast-specific genes [91]. The fibroblast-to-iN induction rate was significantly hindered by *Dnmt3A* inhibition, which suggested that DNA methylation should be monitored in conjunction with histone modifications to improve understanding of cell reprogramming in mature astrocytes as well as in general.

### 3.3. Metabolic Transition

It has been established that a particular cell type can be operationally defined by its unique profile of metabolic pathways. As examples, astrocytes are mainly dependent on anaerobic glycolysis to produce ATP (albeit with the ability to deploy oxidative phosphorylation when in need), whereas neurons utilize oxidative phosphorylation for ATP generation to meet their much higher metabolic demands [96]. Thus, during direct AtN reprogramming and conversion, astrocytes appeared to undergo a phasic metabolic transition to experience an early increase in metabolic demands, which was followed by a metabolic switch to turn on oxidative mechanisms as a possible pre-requisite for “acting” like neurons [97]. Some published data have supported this rationale in that experimental upregulation of Hexokinase 2 (*HK2*) and lactate dehydrogenase (*LDHA*) (i.e., glycolysis genes) blocked the AtN induction process [97,98].

It is worth noting that in iPSC reprogramming, the metabolic transition occurs gradually; in contrast, this switch in direct cell reprogramming and conversion appeared instantaneous, which triggered substantial oxidative stress, including severe lipid peroxidation to cause ferroptosis of the newly induced cells [97]. Therefore, the metabolic transition-induced oxidative stress represents not only a key hazard but also a potential trigger to induce pan-expression of protein markers (i.e., the cell stress response). Addressing the latter concern requires adding housekeeping genes as controls to avoid misreading the non-selectively expressed “neuronal markers” in the post-induction cells, especially for those that experienced non-lethal oxidative stress [99].

Russo et al. (2021) reported that the mitochondrial proteome differed substantially between astrocytes and neurons [100]. This difference represents another major barrier to mature cell phenotypic change because an increase in mitochondrial activity is apparently required before any major metabolic switch can occur and be physiologically sustainable in a cell. Mitochondrial proteins such as sfxn5 and Cpox were abundant in astrocytes, whereas high levels of glutaminase, ATP citrate lysate, and Prdx2 were features of neurons [100,101]. It was reported that ectopic expression of *Ascl1* caused a significant but late onset decrease in astrocyte-enriched proteins but a faster increase in neuron-enriched proteins (i.e., in 5–7 days post-vector injection) [100]. On the other hand, it remains unclear how a single TF can trigger such a quantitative and qualitative metabolic switch in the starting cell. To the best of our knowledge, studies investigating the metabolic mechanisms, particularly in the context of direct cell reprogramming or conversion, have been sparse and incomplete.

### 3.4. Other Potential Therapeutic Effects Derived from the Process of AtN Conversion

**Figure 3:**
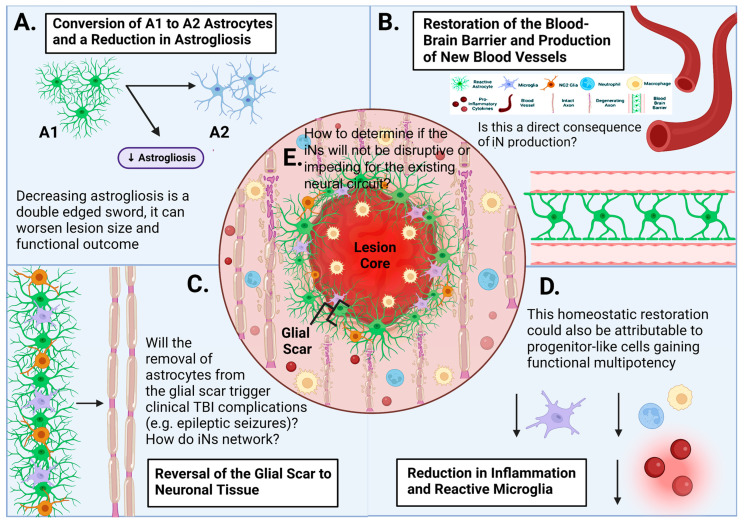
Other therapeutic effects proposed to occur as a result of treatment with AtN protocols. (**A**) An overview of the post-injury microenvironment following TBI or SCI. The acute injury site consists of the activated astrocytes and microglia, infiltration of red blood cells (containing oxidants of Fe^2+^/Fe^3+^), white blood cells, and other immune cells, which jointly trigger oxidative stress/damage and neuroinflammation. These and other secondary injury events lead to reactive astrogliosis and formation of the glial scar, axonal degeneration, and breakdown of the blood–brain/spinal cord barrier (BBB/BSB). (**B**) AtN (astrocyte to neuron) induction has been reported to restore the BBB/BSB. (**C**) AtN protocols were shown to be capable of reversing the glial scar to neural tissue. (**D**,**E**) Direct AtN reprogramming and conversion regimens were reported to exert homeostatic effects of lessening the detrimental inflammatory response and microglial activation post-injury or disease. However, it remains unclear which principle or mechanism can be used to determine if the iNs will be beneficial for the existing or spared neural circuit. Note: real and potential issues and queries with these claims are presented as comments or questions in (**A**–**E**) and discussed in the respective review sections.

In recent years, several in vivo studies exhibited that in addition to the production of iNs, AtN reprogramming and conversion procedures may have other impacts on the local post-injury or degenerating microenvironment, which might be capable of ameliorating the secondary injury cascade in traumatic brain or spinal cord injury (TBI or SCI) (Figure 3). Following injury, astrocytes become reactive, as characterized by hypertrophic morphology and upregulation of proteins such as GFAP and proinflammatory cytokines, in addition to abnormal cell division. These reactive astrocytes have been generally classified into two categories, A1 and A2, based on their functional and gene expression profiles [102]. A1 reactive astrocytes secrete secondary injury factors to escalate reactive astrogliosis and neuronal and oligodendrocyte death, and are implicated in the maintenance of chronic pain [103]. In contrast, A2 reactive astrocytes promote neuronal survival, neuronal network support, tissue repair, and beneficial neuroplasticity (e.g., neural network remodeling) [103,104].

It was shown that reactive astrocytes might be converted into iNs with the ectopic expression of *NeuroD1* in a murine cortical stab wound model; briefly, prior to the conversion into iNs (~3 dpi), reactive astrocytes first entered an intermediate state that resembled A2 astrocytes [105]. At that timepoint, real-time reverse transcription-polymerase chain reaction (rRT-PCR) revealed that A1-associated genes, which had been upregulated 300–900 folds following injury, were significantly downregulated, reducing the overall degree of reactive astrogliosis (semi-quantified by GFAP immunohistochemical signal level). The approach also reduced the expression of chondroitin sulfate proteoglycans (CSPGs), molecules that were shown to have the potential to impede neural repair. A later study using *SOX2* to reprogram NG2 glia (residential glial progenitor cells) into iNs in a SCI model exhibited that the conversion treatment mitigated both astroglial scar volume and surface area [106]. Importantly, to date, there have been no investigations that have tried to determine whether these effects are AtN conversion procedure or TF-specific and the underlying mechanisms.

In a stab wound model, *NeuroD1* induced AtN conversion with high efficiency (90.6 ± 5.2%) and decreased the number of reactive microglia to impede neuroinflammation, resulting in improvement of angiogenesis and restoration of the BBB [23]. The authors postulated that these benefits were attributable to a reduction in pro-inflammatory cytokine release and restoration of the glia to neuron ratio. It was concluded that *NeuroD1*-mediated AtN conversion ignited a new microenvironment surrounding the injury site by transforming what was initially an inhibitory environment into one that was more permissive for neural repair; such an environment might enhance survival and networking of iNs [23].

However, these conclusions were made without addressing some critical questions. For example, since the conversion formula had a reported efficiency of >90%, it is imperative to investigate whether the removal of large numbers of astrocytes from a given glial scar region would trigger more astrocytes to proliferate or cause further dysfunction in the reactive astroglial wall since dramatic changes in astrocyte numbers proximal to the injury epicenter can cause major TBI complications (e.g., synaptic abnormalities, epileptic seizures, etc.; Figure 3) [107,108,109].

These reported beneficial effects could be alternatively produced by stressed cells that manifest progenitor-like features (i.e., possessing functional multipotency to produce trophic factors, exosomes, etc., and to form gap junctions) [110]. Indeed, the improvements in functional recoveries resulting from a multimodal NSC or mesenchymal stromal stem cell (MSC) implant in SCI models were determined not attributable to neuronal replacement through NSC-to-neuron differentiation or MSC-to-neuron transdifferentiation, but instead to the homeostatic effects of donor cells that mitigated the secondary injury events, ameliorated neuroinflammation (including reactive astrogliosis), augmented serotonergic innervation and angiogenesis, and reactivated the spared neural circuits [111,112].

In corroboration with this analysis, it was shown that induction by *Neurog2* via the TRANSCre-DIONE system (a split Cre system under the control of two promoters) produced new MNs and improved locomotor recovery as quantified by the Basso Mouse Scale in a T10 compression SCI model [113]. However, motor neurons around T10 do not directly participate in operating hindlimb locomotion, suggesting that the AtN-generated iNs, per se, likely did not play a major role. Another study exhibited that *NeuroD1* lentivirus injections improved both rotarod and corner test scores in a rodent stroke model [30], which based on neurobiology mechanisms could be caused by different components in the sensorimotor system. Noticeably, the inclusion of behavioral data, in general, has been sparse in AtN induction research using adult animal models, with most reports only presenting data on iNs (Table 2). It is therefore pivotal for future research endeavors to systematically test key alternative hypotheses before more definitive conclusions can be reached.

## 4. Common Issues concerning Cell Phenotype Reprogramming and Conversion Protocols

### 4.1. Control of Specific Subtypes of iNs

One of the main issues with in vivo AtN conversion or reprogramming is the current lack of control over the iN subtype. In order for this strategy to become truly validated, standardized protocols for safely achieving specific neuronal subtypes (i.e., the functionally, molecularly, and/or morphologically distinct types of neurons) in vitro and in vivo in both normal and abnormal conditions must be established. The challenge to this necessity has been highlighted by the divergent cellular identities resulting from studies manipulating the exact same TF(s) in the same starting cell type [35,114]. Theoretically, for an AtN protocol to produce a subtype of neurons, an effective reprogramming or conversion protocol must judiciously modify the epigenetic and genetic regulations of the starting cells, so they can re-enter an earlier ontogenic state where sufficient levels of plasticity remerge (or are directly activated) to permit a metabolic and phenotypic switch to become the pre-determined cell type (e.g., dopaminergic neurons). Clinically, these processes must take place in active interaction with a targeted brain or spinal cord region, which is under the influences of the disease or trauma state, age, gender, and likely any previous or existing therapeutic interventions for the individual. Therefore, the investigation of the combinational conditions, dosage and time course of TFs, and small molecules or miRs requires performing systematically designed and specifically controlled studies before tangible conclusions may begin to surface (for limitations identified in already published studies, see Table 2).

To specify, *Ngn2-* or *Ascl1*-mediated cortical gray matter astrocyte-to-motor neuron induction was reported to require co-delivery of the nuclear receptor-related protein 1 (*Nurr1*) to obtain a higher yield [41]. *Nurr1* typically promoted a dopaminergic phenotype [26], not glutamatergic neurons (e.g., the cortical motor neuron). Further, Nurr1 could affect the nuclear factor kappa B (Nf-ĸB) promoter in the CNS to modulate inflammatory genes. Considering that AtN processes are highly sensitive to inflammatory cytokines and reactive oxygen/nitrogen species (ROS/RNS) [97], these impacts might partially underlie the reported *Nurr1*-based procedures for AtN induction. Conceivably, the Nf-ĸB-related functional consequences could be partly responsible for the reported neural recovery benefit following the AtN induction treatment in a rodent cortical stab wound model [41].

Evidently, this line of work can be strengthened by conducting more in vitro studies focusing on exploring specific mechanisms underlying the specificity of neuronal and neuronal subtype reprogramming/conversion in post-developmental cells. For this purpose, recent advances in single-cell transcriptome analysis have effectively enhanced the field’s ability to profile key epigenetic requirements to drive progenitors toward developing into a specific neuronal subtype. The AtN investigations may become more tangible if performed first in vitro by deploying conventional 2D and more innovative 3D cell culture models including the organoid assay system [115], as well as the mitochondrial proteome assessment [100,101]. Importantly, it remains urgent for the ongoing investigations to utilize multidimensional parameters (e.g., neurotransmitter synthesis, storage, secretion, and metabolism; synaptic, cellular, and neurocircuit functional outcomes; and conditions to maintain the induced neuronal markers and neuron-like behaviors), in addition to the conventional biomarkers to analyze outcomes of direct AtN reprogramming and conversion formulas.

### 4.2. Age of Starting Cells

Data from iPSC generation research suggest that the induction potential decreases with the aging of donor cells [116,117]. In the context of AtN conversion, Liu et al. (2015) showed that both the conversion efficiencies and ratio of Glutamatergic: GABAergic phenotypes achieved with AAV-*Ascl1* were similar between young (P12–P15) and young adult (P60) wild-type (WT) mice (Table 2) [29]. There was, however, no assay that was performed to determine the maturity degree of the pre-transfection astrocytes in P12–P15 mice and P60 mice. Hence, more precise analyses need to be carried out to compare the conversion rate of developmental cells with that of truly aged cells (e.g., ~2-year-old rodent cells; see below) because aging is the biggest risk factor for cells to accumulate unrepaired genetic mutations and for individuals to encounter neurodegenerative diseases, stroke, and certain types of neurotrauma [118,119,120].

Interestingly, the number of iNs post *NeuroD1*-induction was higher in a 14-month-old mouse model of Alzheimer’s disease (5xFAD strain), compared with either the 7-month-old 5xFAD or the WT control group. The difference was attributed to the augmented number of reactive astrocytes in the older diseased animals, which might be more responsive to the vector treatment [121]. The study, however, presented no data about the nonspecific expression of iN markers and phenotypic stability of the detected iNs. These questions are important because neuroinflammation can trigger pan-upregulation of protein markers, and iNs derived from fibroblasts of Alzheimer’s disease patients were recently found to display age-dependent instability in their neuron-like features [122]. It has been suggested that when fibroblasts, astrocytes, and neurons age or suffer inflammation and disease, they undergo “epigenetic erosion” which refers to a gradual transition from the cell’s phenotypic identity towards a more abnormal state with immature and less differentiated features due to stress/repair-related substantial changes in transcriptomic, mitochondrial, and nuclear pore properties [123,124]. Senescent astrocytes, which accumulate in the aged brain, exhibited telomere attrition, high levels of oxidative stress, and inflammatory responses [125]; many epigenetic hallmarks of cellular aging were found to be preserved in the iNs, triggering hypo-mature, dysfunctional, and stressful behaviors in iNs generated from aged starting cells [122,126]. Therefore, more research is required to not only verify the neuronal validity of the iNs but also assess the impact of aging and specific pathological conditions (e.g., oxidative stress, neuroinflammation, etc.) on the effects of the AtN reprogramming or conversion procedures and cellular results (Table 2).

### 4.3. Astrocytic Regional Identity

Both endogenous astrocytes and neurons manifest genetic, epigenetic, and morphological differences depending on their location within the CNS, which corresponds to the functions required by the local and systemic neurocircuits [127,128]. To rebuild damaged, degenerated, or diseased neural networks, acquiring new neurons such as iNs of the correct regional identity with precise functional capacity is of paramount importance. It has been postulated that because both neurons and astrocytes in the CNS are derived from the same germinal zone, they may share region-specific transcriptional and epigenetic characteristics. Pilot studies using clonal analysis uncovered the existence of common nucleus-specific progenitors for neurons and astrocytes; these traits were maintained following *Neurog2*-based AtN induction to confer regional specification in the iNs [129].

Environmental cues have long been established as another major contributing factor that governs stem or progenitor cell proliferation, migration, phenotypic differentiation, maturation, and stability including network engagement and performance [130,131]. Mattugini et al. (2019) reported that overexpression of *Neurog2* and *Nurr1* in reactive astrocytes located inside the cortical gray matter of a murine full-range cortical stab wound model generated iNs carrying lamina-specific features [41]. In contrast, no iNs were formed in the white matter. The data were used to suggest the importance of region and tissue specificity of astrocytes, which could be tapped for iN generation. Unfortunately, none of the mechanisms of cell conversion/reprogramming TFs so far published have been probed to explain the AtN data concerning astrocyte regional identity.

## 5. Cell Lineage Tracing

An ongoing subject of considerable focus is whether the detected iNs are in fact truly derived from the vector-transfected astrocytes. As an example, despite a myriad of studies claimed successful AtN conversion in different disease or injury models via using both ectopic expression of *NeuroD1* and *Ptbp1* knockdown [34,35], there has now been a surge in the number of papers disputing these reports on the ground of data interpretation [132,133,134]. With regards to *Ptbp1* knockdown, studies have reported that (1) *Ptbp1*-based AtN protocols failed to produce iNs in the striatum and substantia nigra of *Ptbp1* knockout mice [132]; (2) only very mild changes in gene expression were observed in the astrocytes following genetic loss of PTBP1 function in both heterozygous and homozygous *Ptbp1^lox/lox^* transgenic mice [135]; and (3) the AAV vector could leak into endogenous neurons causing mislabeled artifacts [136].

One of the comments touched on the possibility that the studies transfected quiescent rather than proliferating astrocytes, which were not as amenable to direct reprogramming or conversion. Furthermore, a recent work added to the analysis of the *Ptbp1* knockdown tactic by generating cell lineage tracing data to show that *Ptbp1* knockdown mediated by AAV-*shPtbp1* and ASO failed to convert either quiescent or reactive astrocytes into iNs [133]. Trying to offer evidence regarding the origin of the iNs, another study undertook a cell lineage mapping approach using *Aldh1l1*-CreER^T2^ mice and showed that the so-called iNs resulting from applying the published protocol [136,137] were indeed adult host neurons that had been mislabeled due to the AAV vector leakage [136]. Considering many major weaknesses demonstrated by the existing AtN protocols, it will be pivotal for the field to utilize stringent cell lineage mapping strategies to verify the identity and the origin of iNs resulting from treatments of published AtN recipes.

## 6. Future Directions

### 6.1. Micro-3D Cell Culture Systems

A major shortcoming of conventional in vitro models (e.g., monolayer cell cultures) is their inability to recapitulate the human brain or spinal cord physiology with regard to the varied cell types [138], complex cell–cell interactions [139], mechanical properties [140], and dynamic fluidic conditions [141]. Micro-3D cell culture systems such as organ-on-a-chip (OOAC) devices and stem-cell-derived organoids have been used as more physiologically relevant models to overcome these problems of the 2D setting, albeit with major deficits in their capacity to factor in the effects of systemic blood circulation, immune regulation, and neural modulation. Aiming to bridge the gap between 2D cultures and in vivo models, both OOAC and 3D organoids, particularly those formed by differentiation of patient-specific iPSCs, are widely used in investigations of neurodevelopment, neurological diseases, and the process of aging [142,143,144,145]. The OOAC is a miniaturized micro-fluidic platform engineered to simulate the 3D micro-environment of a human organ or tissue [146]. Advanced microengineering techniques can recapitulate and regulate some structural and functional parameters including sheer stress, concentration gradients, cell patterning, nutrient supply, and waste removal [13]. Alternatively, 3D brain organoids are stem-cell-derived multi-cell type microtissues that self-assemble into cytoarchitectures that reflect certain characteristics of specific brain regions, offering an assay platform to evaluate how cells respond in vitro as they may partially do in vivo [144,147].

Leveraging and applying these micro-physiological systems to direct AtN reprogramming and conversion research, especially if used in conjunction with single cell transcriptomics, live imaging, CRISPR-cas9 gene editing, and/or optogenetics [148,149,150], may provide deeper insight into the transcriptomic, epigenetic, and genetic underpinnings of the actions of pioneer and reprogramming factors. Moreover, they should allow for better assessments of what additional factors in the non-transfected cells and surrounding ECM may be involved in enabling the detection of conventional biomarkers for describing iNs and host neurons. This could be valuable for OOAC models, where the correct proportion of neurons, astrocytes, and oligodendrocytes (i.e., the neuron-to-glia ratio) may be approximated. These models also permit the capture of transition phases, which is critical for understanding the nature of the transient “plastic stage” that astrocytes may pass through during the assumed reprogramming or conversion, allowing access to data generally inaccessible in animal models. Combining OOAC devices with human iPSCs and 3D bioprinting has already been exploited to create personalized brain chips and organoids [151], which holds potential for verifying available AtN protocols.

It is important to understand that 3D organoids reflect an immature fetal-like neural cell cluster [152]. When trying to translate findings from such a system, one must consider all differences between specifically formulated organoids and in vivo AtN induction in the adult or aged brain or spinal cord with pathophysiological conditions [153]. Another limitation of these micro-3D cell culture systems is that they do not model the systemic immune response, which is a vital component affecting not only viral vector-based interventions but also the injured or diseased environment in vivo as neuroinflammation is ubiquitous in CNS injuries and diseases [154,155]. Hence, emerging studies have started incorporating microglia into 3D cultures [156,157,158,159]. Other plausible improvements to enhance the analysis of direct AtN reprogramming and conversion research include (1) utilization and optimization of 3D assays [160]; (2) the generation of 3D model-specific robotic assays and automatic handling systems to strengthen data tangibility [161]; and (3) the generation of assembloids via joining organoids from different brain or spinal cord regions to reveal the impact of existing AtN procedures on the glial and neuronal network [162].

### 6.2. Spatial Biology

Spatial biology describes how transcriptional dynamics are influenced by the spatial context of cells. Its investigation is mostly done through methods that combine immunofluorescence and high-plex gene expression assays in cell location and interaction-specific manners [163]. Thus, spatial biological data can quantify biomarker expression at single cell resolution (e.g., using photocleavable tags which can be harvested for next-generation sequencing) for 3D population or subpopulation reconstructions [164] to shed light on how cells are organized and interact in a targeted local micro-environment at a level unobtainable with bulk sequencing. Understanding how gene expression varies in a 3D context helps to interpret functional and biological adaptations occurring in the cell in health, disease, therapeutic, and/or investigative conditions including direct AtN induction procedures. Because the brain is a highly organized and structured organ, spatial biology-based probing can yield novel information regarding the tangibility of iN data obtained from the micro-3D and in vivo models. These technologies will also help to disentangle the complex interactions between iNs and host neural cells and the surrounding environment in either the healthy or diseased state [165].

### 6.3. Other Delivery Methods

The majority of studies attempting direct AtN reprogramming and conversion in vivo administered TFs and/or other factors via stereotaxic microinjections into the target region of the brain or spinal cord (Table 2). However, this approach is unlikely to be translated into the clinic due to safety considerations. Conversely, if systemic delivery would become a viable option, the AtN agents must possess qualities including a sufficiently long plasma half-life, the ability to cross the BBB/BSB and cell membrane, and efficient endosomal escape mechanisms [166,167].

To date, no published major studies of AtN conversion have examined the outcome and effect of deploying tactics of smart drug delivery such as microbubbles for sonoporation [168], gold nanoparticles [169,170,171], and cell-penetrating peptides (CPPs, otherwise known as protein transduction domains) [172,173].

## 7. Additional Discussions and Concluding Remarks

In reputable English dictionaries (i.e., Merriam-Webster and Oxford Languages, ©2023), conversion is defined as the process of changing from one form to another, or the fact of changing one’s religion or beliefs. Reprogramming refers to reworking out a sequence of operations to be performed by a mechanism such as a computer. In biology, conversion is traditionally used to describe metaplasia (i.e., the irreversible conversion of one differentiated cell or tissue type into another) [174]. Reprogramming indicates removal and/or alteration of epigenetic marks during development or changing one cell fate to another, particularly implying transformation of a mature differentiated cell into a less-committed precursor [175]. Overall, conversion indicates a decisive process with both pre-existing subject/state and post-change outcome clearly defined (and likely known steps to attain the outcome). Conversely, reprogramming describes consecutive modification of epigenetic marks to affect cell development or phenotype, a journey where reprogramming and cell lineage alteration may simultaneously and/or consecutively take place. Conceivably, the ongoing interchangeable use of “direct conversion” and “direct reprogramming” may have been subtly casting hurdles (e.g., preventing generation of more precise nomenclatures or subclassification of mechanisms underlying varied AtN protocols) and even causing confusion regarding what exactly is required to alter a somatic cell’s phenotype and maintain the new cell type in an adult animal. Therefore, for this paper per se, we have tried to distinctively describe direct conversion and direct reprogramming as per the experimental methods utilized to start the process. The anticipation is that our introduction of a recipe-framed subclassification system, which is factually built following prospective logic analysis principles, may likely encourage development of more innovative investigations to qualitatively improve this line of research.

There remain several fundamental theoretical questions and experimental barriers that forestall drawing tangible conclusions regarding the nature of the iNs that were identified in situ following commonly reported direct AtN reprogramming and conversion interventions in adult animal models. Therefore, it is crucial for the field to come up with scientifically defined theoretical frameworks explaining (1) how/why mature astrocytes in situ may become vulnerable to entering phenotypic switch upon receiving actions of one or a few TFs; (2) how/why networked mature astrocytes after direct AtN induction treatment may develop proper dendrites and axons to join functional and/or malfunctioning neuronal circuits; and (3) how/why the regional specific ratio between astrocytes, oligodendrocytes, microglia, and neurons may be maintained or restored under the effects (including stress) of the AtN reprogramming and conversion agents.

On the bench side, it appears imperative for the research studies to concentrate on validating key outcomes of direct AtN induction using advanced in vitro cell culture and disease/trauma modeling systems before experimenting with such protocols in animals in vivo. Collectively, the findings of this review suggest that for the field to move forward, future investigations need to focus on (i) improving the selectivity of vectors for astrocytes (i.e., the starting cell) in vitro and in vivo through developing new technologies that are able to distinguish between neurons, astrocytes, microglia, and possible iNs to avoid mislabeling and mis-characterization; (ii) establishing new standardized criteria for transcriptional, epigenetic, functional, and metabolic assessments to define what constitutes the effect(s) of a published AtN protocol-based treatment on mature differentiated astrocytes, oligodendrocytes, microglia, and neurons in vitro before testing the formula in vivo; and (iii) optimizing experimental designs to investigate alternative hypotheses.

## Supplementary Materials

The following supporting information can be downloaded at: https://www.mdpi.com/article/10.3390/cells12040618/s1, Table S1: Abbreviations and Acronyms; Table S2: Common Markers or Agents for Detecting Phenotype or Function of Neurons and Induced Neurons (iNs).
