# Supplementary material for: Direct Cell Reprogramming and Phenotypic Conversion: An Analysis of Experimental Attempts to Transform Astrocytes into Neurons in Adult Animals
Source: Cells. 2023 Feb 14;12(4):618. doi: 10.3390/cells12040618 (PMC9954435; doi:10.3390/cells12040618)
Supplement: Supplementary file 1 [file cells-12-00618-s001.zip › cells-2096074-supplementary.pdf]

Supplementary Materials

Table S1. Abbreviations and Acronyms.

**Abbreviation/Acronym**	
**5XFAD Mice**	The 5XFAD transgenic mice overexpress mutant human amyloid beta (A4) precursor protein 695 (APP) with the Swedish (K670N, M671L), Florida (I716V), and London (V717I) Familial Alzheimer's Disease (FAD) mutations along with human presenilin 1 (PS1) harboring two FAD mutations, M146L and L286V
**AAV**	Adeno-associated virus
**AAVsh-PTBP1**	Targeting PTBP1 with AAVshRNA
**ALN**	Ascl1, Lmx1a, and Nurr1
**A-MHC**	Alpha-myosin heavy chain
**AP**	Action potential
**Ascl1**	Achaete-scute homolog 1
**ASO**	Anti-sense oligonucleotide
**AtN**	Astrocyte to neuron
**ATP**	Adenosine triphosphate
**BAF53a/ACTL6A** (a subunit of the Brg/Brm-associated factor [BAF] complex/Actin Like 6A)	An actin-related protein involved in transcriptional activation and repression of select genes by chromatin remodeling (i.e., alteration of DNA-nucleosome topology)
**BAM**	Brn2, Ascl1, Myt1l
**BBB**	Blood brain barrier
**BDNF**	Brain-derived neurotrophic factor
**Brn2**	POU-homebox-domain-containing octamer-binding transcription factors Pou3f3/Brn1 and the closely related Pou3f2/Brn2.
**BTB**	Broad complex, tramtrack, and Bric-a-Brac
**Cas-13d**	CRISPR associated protein-13d
**cC**	Cell capacitance
**CCM**	Current clamp mode
**CHAT**	Choline acetyltransferase
**CHIR99021**	An inhibitor of glycogen synthase kinase-3 (GSK-3)
**CNS**	Central nervous system
**CpG Methylation**	DNA methylation of CpG islands. A CpG island is defined as a 200-bp region of DNA with a cytosine-guanine (C-G) content higher than 50% and the cytosine is 5′ to the guanine nucleotide (multiple methylated CpG sites in CpG islands of promoters result in stable silencing of genes)
**Cpox**	Coproporphyrinogen oxidase
**CreER^{T2}**	Encoding the fusion protein of Cre recombinase being fused to a triple mutant form of the human estrogen receptor that binds tamoxifen (4-hydroxytamoxifen [OHT]) for activity
**Cre-LoxP System**	The 38-kDa DNA recombinase produced from cre (cyclization recombinase) gene of bacteriophage P1 recognizes the specific DNA fragment sequences of loxP (locus of x-over, P1) site and mediates site-specific deletion of DNA sequences between two loxP loci

**CRISPR**	Clustered regularly interspaced short palindromic repeats
**CTIP2**	COUP TF1-interacting protein 2
**DAPT**	(2S)-N-[(3,5-Difluorophenyl)acetyl]-L-alanyl-2-phenyl]glycine 1,1-dimethylethyl ester, an inhibitor of γ-secretase and notch signaling
**DAT**	Dopamine transporter
**dcas9**	Dead Cas9
**dECM**	Decellularized extracellular matrix
**DLX3**	Distal-less homeobox 3
**dMP**	Depolarized membrane potential
**Dnmt3a**	DNA Methyltransferase 3 Alpha
**dpi**	Days post induction
**DREADD**	Designer receptors exclusively activated by designer drugs
**emDA**	Excitable midbrain dopamine neurons
**FAD**	Familial Alzheimer's disease
**FDA**	Food and drug administration
**FUGW Lentiviral Vector**	A self-inactivating and replication-incompetent lentiviral vector that carries the human ubiquitin-C promoter driving a GFP reporter gene
**GAD1**	Glutamate decarboxylase 1
**GAP43**	Growth associated protein 43
**GC/mL**	AAV as genome copies (gc) per mL
**GFAP**	Glial fibrillary acidic protein
**GFP**	Green fluorescent protein
**GLUT2**	Glucose transporter 2
**H3K27 Acetylation**	Acetylation of the lysine residue at N-terminal position 27 of the DNA packaging protein histone H3 protein, which is an active enhancer mark
**HK2**	Hexokinase 2
**I-BET151**	an inhibitor of bromodomain and extra-terminal domain (BET) proteins
**iCMs**	Induced cardiomyocytes
**iN**	Induced neuron
**iNSCs**	Induced neural stem sells
**IOC**	Inward and outward Na/K current
**iPSCs**	Induced pluripotent stem cells
**iR**	Input resistance
**ISL1**	Islet1 is an insulin gene enhancer protein that is a LIM homeodomain transcription factor (LIM comprises the initials of Lin11 and Mec-3 in C. elegans and Isl-1 from rat)
**ISX9**	Isoxazole 9
**JAK/STAT**	Janus kinase/signal transducers and activators of transcription pathway
**KLF**	Krüppel-like factor
**KMT2B**	Lysine methyltransferase 2B is a protein coding gene
**KRAB**	Krüppel-associated box
**LDHA**	Lactate dehydrogenase
**LDN193189**	A potent inhibitor of the bone morphogenetic (BMP) pathway (also called dihydrochloride)
**LHX3**	LIM homeobox protein 3, a LIM-homeodomain transcription factor
**LMX1A**	LIM homeobox transcription factor 1 alpha
**MAFA**	MAF BZIP transcription factor A

**MAP2**	Microtubule associated protein 2
**MC**	Membrane capacitance
**mCherry**	A member of the mFruits Family of monomeric red fluorescent proteins (mRFPs), which was derived from Discosoma sea anemones
**Mef2c**	MEF2C myocyte enhancer factor 2C
**MI**	Myocardial infarction
**miR**	Micro-RNA
**miR***	The * has previously been used to refer to the passenger strand, which is thought to be fully degraded. More recent evidence suggests that miR*s can display functionality and play complementary roles to their related miRs
**MNX1**	Motor neuron and pancreas homeobox 1
**MP**	Membrane potential
**mRNA**	Messenger RNA
**MSCs**	Mesenchymal stromal stem cells
**MSN**	Medium spiny neurons
**MYL9**	Myosin light chain 9
**Myt1**	Myelin transcription factor 1
**Myt1l**	Myelin transcription factor 1-like protein.
**MΩ**	Membrane resistance
**Nerfin 1**	Nervous fingers 1, a zinc finger transcription factor
**NeuN**	Neuronal nuclei
**NeuroD**	Neuronal differentiation (gene or factor)
**NF-κB**	Nuclear factor Kappa B
**NG2 Glia**	Nerve/glial antigen 2 Glia
**NgN**	Neurogenin
**Non-CpG Methylation**	DNA methylation at cytosines followed by adenine, thymine, or another cytosine instead of a guanine. An epigenetic signature of physiological mature neurons, and play a key role in gene expression regulation
**NPCs**	Neural progenitor cells
**Nurr1**	Nuclear receptor related protein 1
**OOAC**	Organ-on-a-chip
**OPCs**	Oligodendrocyte precursor cells
**Pax6**	Paired box protein Pax-6
**PBAEs**	Poly(β-amino esters)
**Pdx1**	Pancreatic and duodenal homeobox 1
**Prdx2**	Peroxiredoxin-2
**PTBP1**	Polypyrimidine tract-binding protein 1
**PTBP1$^{lox/lox}$ Mice**	These transgenic mice carry loxP sites that flank the promoter and 1st exon of PTBP1
**P300**	Histone acetyltransferase
**rAP**	Repetitive action potential
**REST**	Repressor element-1 silencing transcription factor
**RFP**	Red fluorescent protein
**RMP**	Resting membrane potential
**RNA**	Ribonucleic acid
**ROS/NOS**	Reactive oxygen species/nitrogen species
**RTMP**	Remodeling TGFβ midbrain protocol
**rtPCR**	Reverse transcription polymerase chain reaction

**rtTA**	Reverse tetracycline-controlled transactivator
**SB431542**	An inhibitor of the transforming growth factor beta (TGF-β), activin and nodal signaling pathways
**SC**	Spinal cord
**SCI**	Spinal cord injury
**SE**	Synaptic event
**sEA**	Spontaneous electrical activity
**sEPSC**	Spontaneous excitatory postsynaptic current
**SeV**	Sendai virus
**Sfnx5**	Sideroflexin 5
**sgRNA**	Synthetic guide RNA
**shRNA**	Short-hairpin RNA
**sIPSC**	Spontaneous inhibitory postsynaptic current
**siRNA**	Small interfering RNA
**sPSC**	Spontaneous postsynaptic current
**S100**	S100 protein family is the largest subgroup of the Ca^{2+} binding EF-hand (helix E-loop-helix F) proteins. S100 refers to their solubility in a 100%-saturated solution with ammonium sulphate at neutral pH
**TBI**	Traumatic brain injury
**Tbx5**	T-Box transcription factor 5
**TET-1**	Ten-eleven translocation methylcytosine dioxygenase 1
**TH**	Tyrosine hydroxylase
**Tuj1**	Beta III tubulin
**UD**	Unpublished data
**VOC**	Voltage clamp mode
**wpi**	Weeks post injury
**WT**	Wild type
**Y-27632**	An inhibitor of the rho-associated, coiled-coil containing protein kinase (ROCK)
**ZFP238**	Zinc finger protein 238
**6-OHDA**	6-hydroxydopamine

Table S2. Common Markers or Agents for Detecting Phenotype or Function of Neurons and Induced Neurons (iNs)

1) Neuronal Markers

Abbreviation	Full Name	Target	Description
**TUJ1**	The Clone Name of the Original Monoclonal Antibody against Beta Tubulin III	Immature neurons	Neuron-specific Class III β-tubulin, marker of early neural differentiation
**NeuN**	Neuronal Nuclei	Mature neurons	A protein (a homologue to the protein product of a sex-determining gene in Caenorhabditis elegans) that is a neuronal nuclear antigen used as a marker for mature neurons
**MAP2**	Microtubule Associated Protein 2	Neuronal differentiation marker (dendrites)	Mainly neurons, some oligodendrocytes, weak signal in neuronal precursor, strong signal after BIII expression
**BIII**	Class III Beta-Tubulin	Early neuronal marker	Microtubule element of tubulin family, possibly expressed in fetal astrocytes as well
**Synapsin I**	Synapsin 1	pre-synaptic terminal marker	Family of neuron-specific phosphoproteins, most abundant on synaptic vesicles, involved in synaptogenesis and neuronal plasticity
**TH**	Tyrosine Hydroxylase	Dopaminergic neuronal marker	Rate-limiting enzyme in synthesis of Dopamine, Norepinephrine, Epinephrine (catecholamines)
**SYN**	Synuclein	Pre-synaptic neuronal protein	Small, soluble proteins expressed primarily in neural tissue. The family includes three known proteins: α-synuclein, β-synuclein, and γ-synuclein. α-Synuclein is a presynaptic neuronal protein that is linked genetically and neuropathologically to Parkinson's disease

2) Agents or Markers for Identifying the Neuronal Function or Subtype of Neurons and iNs

Abbreviation	Full Name	Effect/Function	Description
**CNQX**	Cyanquixaline (6-cyano-7-nitroquinoxaline-2,3-dione)	Blocking glutamatergic neuronal function	Competitive AMPA/kainate receptor antagonist
**Bicuculline**	Bicuculline	Blocking GABAergic neuronal function	Light-sensitive competitive GABA-A receptor antagonist, inhibits GABA binding
**DDC**	L-DOPA Decarboxylase	Dopaminergic neuronal marker	Enzyme in conversion of L-DOPA to dopamine and L-5-hydroxytryptophan to serotonin
**SLC6A3**	Solute Carrier Family 6 Member 3	Dopaminergic neuronal marker	Gene encoding dopamine transporter (DAT)
**FOXA2**	Forkhead Box Protein A2	Dopaminergic neuronal marker	Transcription factor controlling differentiation and development of dopaminergic neurons
**EN1**	Engrailed Homeobox 1	Dopaminergic neuronal marker	Transcription factor specifically expressed in adult midbrain dopaminergic neurons soon after differentiation
**SLC18A**	Solute Carrier Family 18 Member A	Neuronal marker	Gene encoding integral transmembrane protein that transports acetylcholine and monoamines (e.g., dopamine, norepinephrine, serotonin, and histamine) into synaptic vesicles
**NR4A2**	Nuclear Receptor Subfamily 4 Group A Member 2	Dopaminergic neuronal marker	Part of orphan nuclear receptor family, key regulator of dopaminergic neuron development

**ALDH1A1**	Aldehyde Dehydrogenase 1A1	Predominantly expressed in the substantia nigra	Retinaldehyde dehydrogenase that catalyzes irreversible oxidation of aldehydes, expressed highly in dopaminergic neurons in the midbrain and is a marker of astrocytic differentiation
**GIRK2 (KCNJ6)**	G-protein-Regulated Inward-Rectifier Potassium Channel 2	Predominantly expressed in the substantia nigra	G-protein inward rectifying K^+ channel that regulates neuronal excitability, expressed regionally in other areas of brain as well
**PBX1**	Pre-B-cell Leukemia Transcription Factor 1	Dopaminergic neuron marker	Homeodomain transcription factor that regulates numerous embryonic processes such as organogenesis and hematopoiesis. Shown to directly activate or repress certain gene expressions, such as Pitx3 that is important to lens formation and promotes dopaminergic neuron development
**Tbr1**	T-box, Brain 1	Neuronal marker	Gene encoding neuron-specific transcription factor in cortex and involved in the differentiation of projection neurons
**DAT**	Dopamine Transporter	Dopaminergic neuron marker	Plasma membrane dopamine transporter
**Glutaminase**	Glutaminase	Glutamatergic neuron marker	Breaking down glutamine to form glutamate, expressed in various cells and mitochondria (glutamate can be converted to α-ketoglutarate to enter The tricarboxylic acid cycle)
**vGlut1**	Vesicular Glutamate Transporter 1	Glutamatergic neuron marker	Glutamate/Proton exchanger that transports glutamate into synaptic vesicles
**vGlut2**	Vesicular Glutamate Transporter 2	Glutamatergic neuron marker	Glutamate/proton exchanger mostly in thalamus, midbrain, and brainstem, also expressed in subset of dopaminergic neurons
**GAD65/67**	Glutamate Decarboxylase 65	GABAergic neuron marker	Glutamate decarboxylase that converts glutamate into GABA (GAD65 is found in

	kD or 67 kD isoform		the synaptic terminals of the neurons as well as in pancreatic cells; GAD67 is throughout neurons)
**vGaT**	Vesicular GABA Transporter	GABAergic neuron marker	Transporting GABA and glycine into synaptic vesicles
**DARPP32**	Dopamine- and cAMP-regulated Phosphoprotein (Mr 32 kDa)	Medium spiny neuron (MSN) marker	Protein kinase a (PKA) substrate that regulates various neuronal processes and has enriched expression in MSN, but not exclusive
